# The expression patterns of different cell types and their interactions in the tumor microenvironment are predictive of breast cancer patient response to neoadjuvant chemotherapy

**DOI:** 10.1101/2024.06.14.598770

**Published:** 2024-06-14

**Authors:** Saugato Rahman Dhruba, Sahil Sahni, Binbin Wang, Di Wu, Padma Sheila Rajagopal, Yael Schmidt, Eldad D. Shulman, Sanju Sinha, Stephen-John Sammut, Carlos Caldas, Kun Wang, Eytan Ruppin

**Affiliations:** 1 Cancer Data Science Laboratory, Center for Cancer Research, National Cancer Institute, National Institutes of Health, Bethesda, MD, USA.; 2 Laboratory of Pathology, Center for Cancer Research, National Cancer Institute, National Institutes of Health, Bethesda, MD, USA.; 3 Women’s Malignancies Branch, Center for Cancer Research, National Cancer Institute, National Institutes of Health, Bethesda, MD, USA.; 4 NCI-Designated Cancer Center, Sanford Burnham Prebys Medical Discovery Institute, San Diego, CA, USA.; 5 Breast Cancer Now Toby Robins Research Centre, The Institute of Cancer Research, London, UK.; 6 The Royal Marsden Hospital NHS Foundation Trust, London, UK.; 7 Institute of Metabolic Science, School of Clinical Medicine, University of Cambridge, Cambridge, UK.; 8 Department of Clinical Biochemistry, University of Cambridge, Cambridge, UK.

## Abstract

The tumor microenvironment (TME) is a complex ecosystem of diverse cell types whose interactions govern tumor growth and clinical outcome. While the TME’s impact on immunotherapy has been extensively studied, its role in chemotherapy response remains less explored. To address this, we developed DECODEM (DEcoupling Cell-type-specific Outcomes using DEconvolution and Machine learning), a generic computational framework leveraging cellular deconvolution of *bulk transcriptomics* to associate the gene expression of individual cell types in the TME with clinical response. Employing DECODEM to analyze the gene expression of breast cancer (BC) patients treated with neoadjuvant chemotherapy, we find that the gene expression of specific immune cells (*myeloid*, *plasmablasts*, *B-cells*) and stromal cells (*endothelial*, *normal epithelial*, *CAFs*) are highly predictive of chemotherapy response, going beyond that of the malignant cells. These findings are further tested and validated in a single-cell cohort of triple negative breast cancer. To investigate the possible role of immune cell-cell interactions (CCIs) in mediating chemotherapy response, we extended DECODEM to DECODEMi to identify such CCIs, validated in single-cell data. Our findings highlight the importance of active pre-treatment immune infiltration for chemotherapy success. The tools developed here are made publicly available and are applicable for studying the role of the TME in mediating response from readily available bulk tumor expression in a wide range of cancer treatments and indications.

## INTRODUCTION

The tumor microenvironment (TME) is a complex and multifaceted ecosystem that encompasses a plethora of cell types including immune cells (such as T- and B-lymphocytes, macrophages, dendritic cells *etc.*), stromal cells (such as fibroblasts, endothelial cells *etc.*), and the extracellular matrix. It plays crucial roles in regulating tumor progression and response to therapy. Emerging evidence suggest the context-specific behavior of the TME as either tumor-suppressive or supportive, presenting an attractive target for therapeutic intervention [1][2]. Numerous studies have elucidated the critical role of TME in mediating response to immunotherapy, however, insights into how the TME is relevant to chemotherapy response remains limited. Here we focus on studying the influence of the TME on chemotherapy response in breast cancer (BC), where neoadjuvant chemotherapy (NAC) is used prior to primary surgery to enable tumor downstaging and increase the likelihood of breast-conserving surgery [3][4][5].

Recent studies have underscored the relevance of the BC TME in determining patient response to chemotherapy. For instance, chemotherapy can induce dynamic changes in the TME that lead to an increase in regulatory T-cells and myeloid-derived suppressor-like cells, calling for a balance in immune population for positive treatment outcomes [6]. Lymphocyte density and tumor infiltrating lymphocytes have consistently been associated with BC patients achieving pathological complete response (pCR) following NAC and anti-HER2 therapy [7][8][9][10][11]. Additionally, multiple studies have reported the active involvement of TME in therapy resistance [12][13][14], with macrophages, endothelial, and BC stem cells promoting chemoresistance [15][16][17], and B-cells and plasma cells displaying varying roles in NAC response [18][19][20]. However, to our knowledge, no study has yet attempted to quantify the contribution and predictive power of each individual cell type in the TME to chemotherapy response in a systematic and rigorous manner. We, therefore, aim to investigate three fundamental research questions: (1) Can we apply a rigorous computational approach to delineate the cell-type-specific transcriptome predictive power in the BC TME from bulk transcriptomics data? (2) Which are the cell types in the BC TME and the corresponding pathways whose gene expression are most predictive of chemotherapy response? (3) Does aggregating the inferred expression of multiple predictive cell types in the BC TME lead to a more accurate predictor?

Current advances in precision oncology have highlighted the utility of artificial intelligence [1][2][21][22][23][24] in guiding treatment using diverse data types such as genomics [1][25][26], transcriptomics [29][30][31][32][33], histopathology [34][35][36][37][38][39], or through multimodal approaches [11][27][28][40][41][42]. However, investigations into neoadjuvant therapy response often encounter limitations like small sample size, treatment heterogeneity, or inadequate capturing of the TME’s complexity, as highlighted by Sammut et al. [11]. In their pioneering work, Sammut et al. presented a novel multiomic predictor of neoadjuvant therapy response in BC [11]. They have leveraged machine learning (ML) to incorporate 34 features extracted from clinical, DNA, RNA, pathology, and treatment information to develop a single model to predict patient response across all BC subtypes and different treatment regimens with high accuracy. Expanding on their groundwork and addressing the limitations, our study aims to decouple the cell-type-specific effects to NAC response in the BC TME from transcriptomics alone by focusing on the HER2-negative patients, where NAC is recommended to increase the likelihood of achieving pCR following surgery, especially in triple-negative breast cancer (TNBC) [49].

To systematically explore the association between the TME and chemotherapy response, we developed a computational framework, **DECODEM (****DE****coupling** **C****ell-type-specific** **O****utcomes using** **DE****convolution and** **M****achine learning).** This framework leverages cellular deconvolution with machine learning to elucidate the association of gene expression of diverse cell types in the TME to patient response to a given treatment. Recognizing that these phenotype effects are intricately regulated by the interactions among relevant cell types within the TME, we further extended DECODEM to **DECODEMi**, where ‘i’ stands for interaction. DECODEMi incorporates the *inferred* cell-to-cell communications to pinpoint the key cell-cell interactions (CCIs) influencing treatment response. As mentioned, we demonstrate the utility of our tools in an example scenario of investigating the role of the BC TME in modulating response to NAC. A recent study by Kester et al. investigated the association of cellular compositions of the TME with patient survival in BC [43], but to our knowledge, the current study is the first to analyze the *cell-type-specific transcriptomic profiles* in the BC TME to objectively quantify their association with treatment response.

Through the application of DECODEM, we illustrate the markedly improved predictive capabilities of cell-type-specific transcriptome over bulk transcriptome, untangling the individual contributions of different cell types in treatment response. Through the application of DECODEMi, we further pinpoint the notable immune interactions among these cell types. Together all these findings point to the necessity of an active pre-treatment immune system to facilitate positive treatment outcome, as supported by Sammut et al. [11]. Therefore, from a methodological perspective, our study provides tools to systematically assess the role of individual cellular components within the TME in anticancer therapy response (DECODEM), and to identify the prominent cell-to-cell communications associated with this response (DECODEMi). These tools will be made publicly available upon publication.

## RESULTS

### Overview of the analysis

DECODEM and DECODEMi developed and presented here are new computational frameworks designed to explore the predictive powers of individual cell types and relevant cell-cell interactions, respectively, within the TME to response to therapy. They harness the prowess of CODEFACS and LIRICS, two recent tools developed in our laboratory [44]. CODEFACS facilitates the extraction of the cell-type-specific expression profiles from bulk gene expression data for each patient, while LIRICS discerns the activity of cell-type-specific ligand-receptor interactions within the patient’s TME. In essence, DECODEM takes the pre-treatment bulk gene expression as input and outputs the quantified predictive power (in terms of AUC and AP *i.e.*, the area under the receiver operating characteristics (ROC) curve and average precision, equivalent to the area under the precision-recall curve, respectively) of each cell type to treatment response. DECODEMi then follows through to extract the most predictive cell-cell interactions among these cell types.

**Figure 1:**
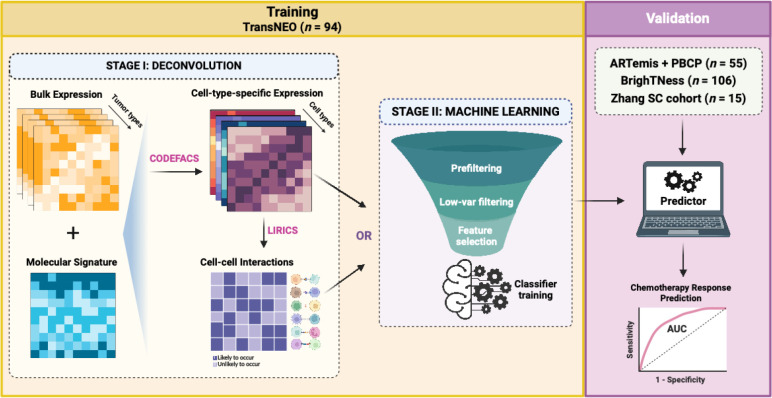
The analysis pipeline, DECODEM and DECODEMi. *First*, we apply CODEFACS on bulk expression to generate nine cell-type-specific expression profiles and further apply LIRICS to infer the cell-cell interactions (CCIs) present in the tumor microenvironment. *Second*, we train a multi-stage machine learning pipeline with either the nine cell-type-specific expression profiles to build nine cell-type-specific clinical response predictors (DECODEM) or the CCI profile to build a CCI-based predictor (DECODEMi). In validation, we deploy the abovementioned first stage to procure the cell-type-specific expression or CCIs and directly feed those as inputs to the trained predictors to evaluate model performance.

Specifically, DECODEM comprises of two sequential steps: *First*, for each bulk expression cohort, it generates nine cell-type-specific expression profiles (as annotated by Wu et al. [45] which were used for molecular signature generation; see details in METHODS: *B-cells*, *Cancer-associated Fibroblasts (CAFs)*, *Cancer Epithelial (malignant)*, *Endothelial*, *Myeloid*, *Normal Epithelial*, *Plasmablasts*, *Perivascular-like Cells (PVL)*, and *T-cells*) in each sample using CODEFACS; *Second*, by leveraging these nine deconvolved expression profiles, it generates nine cell-type-specific predictors of clinical response (responders vs. non-responders *e.g.*, pCR vs. residual disease) using a multi-stage ML pipeline (Figure 1). Within this pipeline, we performed rigorous feature selection to keep the top ‘*m*’ genes per cell type (2 ≤ *m* ≤ 25; the upper limit is chosen to avoid overfitting and facilitate faster runtime) before feeding their expression into an unweighted ensemble classifier consisting of four ML algorithms: regularized logistic regression, random forest, support vector machine, and XGBoost (see details in METHODS). The pipeline was trained with repeated three-fold cross-validation (CV) to optimize model hyperparameters. Additionally, we built a baseline predictor with bulk expression using the same ML pipeline to serve as a control, comparative rod.

To assess the performance of DECODEM and evaluate the contribution of each cell type, we analyzed bulk transcriptomics data from three breast cancer cohorts with different ratios of responders to non-responders (R:NR) to NAC (Supplementary Figure 1) in two scenarios: (a) cross-validation with an additional five-fold CV on the TransNEO cohort (*n* = 94, R:NR = 22:72), recently published by Sammut et al. accompanied by a multiomic clinical response predictor [11], and (b) external validation on the ARTemis + PBCP (*n* = 55, R:NR = 8:47) and BrighTNess (*n* = 106, R:NR = 65:41) cohorts [46][47]. TransNEO and ARTemis + PBCP included all major BC subtypes, among which we focused on the HER2-negative patients [11][46] (Supplementary Figure 1B–C), whereas the BrighTNess cohort included only TNBC patients from arm B of the trial, treated with a combination of paclitaxel and carboplatin with a placebo for veliparib [47]. Moreover, we established DECODEM’s generalizability to single-cell transcriptomics through external validation on an independent cohort from Zhang et al, which consisted of 15 TNBC patients who received either paclitaxel alone (R:NR = 3:3) or in combination with atezolizumab, an anti-PD-L1 agent (R:NR = 4:5) [48]. Notably, we considered the Sammut et al. predictor built with 18 extracted features from clinical and RNA-seq data (e.g. not including the DNA-seq data) as a comparative rod, which achieved an AUC of 0.88 in CV and 0.89 in validation with ARTemis + PBCP [11]. This predictor outperformed a baseline predictor built with seven clinical features including ER status and HER2 status, which are routinely used to guide clinical decisions [49].

To investigate the effect of incorporating the expression profiles of multiple predictive cell types together, as indicated by our third question, we applied two extensions to DECODEM: (i) append the cell-type-specific expression profiles for two and three most predictive cell types together to train a multi-cell-ensemble ML pipeline, and (ii) extend to DECODEMi to incorporate the inferred CCIs to train a CCI-based ML pipeline (Figure 1; see details in METHODS). The former model maximizes the predictive performance by leveraging the complementary information from different cell types while the latter identifies the most predictive cell-to-cell communications for clinical response, thereby providing deeper insights to the underlying mechanism.

### Gene expression of immune and stromal cells predicts response to neoadjuvant chemotherapy as accurately as the expression of malignant cells

**Figure 2:**
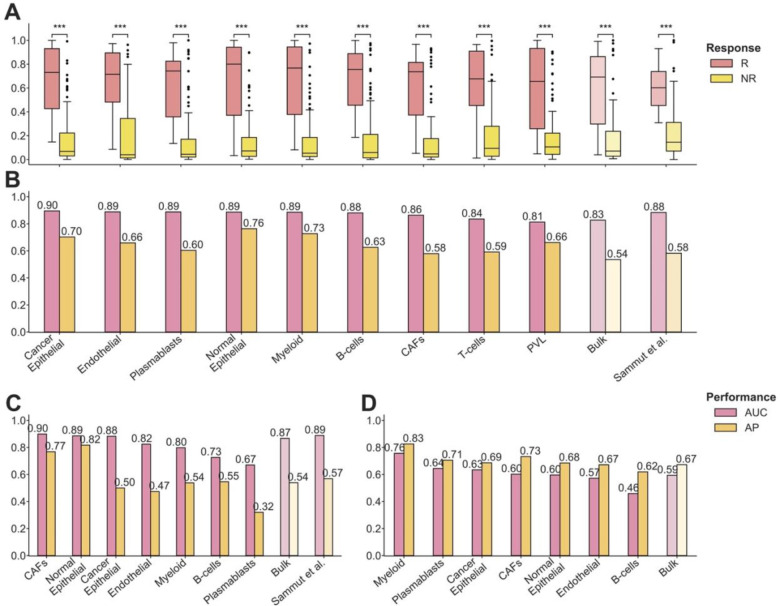
The prominent cell types mediating chemotherapy response in breast cancer tumor microenvironment. **A-B.** Comparison of prediction scores (**A**) and model performance (**B**) in cross-validation with TransNEO (*n* = 91) for nine cell-type-specific, bulk, and Sammut et al. predictors. R and NR stand for responders and non-responders. The differences between the prediction scores were computed by using a one-tailed Wilcoxon rank-sum test. AUC and AP stand for the area under the receiver operating characteristics curve and average precision (equivalent to the area under precision-recall curve), respectively. Cell types are ranked by their AUC values in a descending order. **C.** Comparison of model performance in external validation with ARTemis + PBCP (*n* = 55) for the seven prominent cell types, bulk, and Sammut et al. predictors. Cell types are ranked by their AUC values in a descending order. **D.** Comparison of model performance in external validation with BrighTNess (*n* = 106) for the seven prominent cell types and bulk predictors. Cell types are ranked by their AUC values in a descending order.

To assess the impact of individual cell types on chemotherapy response, we applied DECODEM to TransNEO in a CV analysis. Our analysis demonstrated significant differences (one-tailed Wilcoxon rank-sum test *p*-value ≤ 0.001) in the resulting prediction scores between responders and non-responders across 91 patients (the patients with available predictions from Sammut et al.), with the identified cell types outperforming bulk and Sammut et al. predictors (Figure 2A). Seven out of nine cell types exhibited markedly enhanced predictive capabilities in identifying responders. Notably, immune cells (*myeloid*, *plasmablasts*, *B-cells*) contribute as prominently as the malignant (*cancer epithelial*) and stromal cells (*endothelial*, *normal epithelial*, *CAFs*) (Figure 2B). Our subsequent analyses, focused on these seven ‘prominent’ predictive cell types, whose abilities to accurately identify true responders to chemotherapy in terms of the odds ratio and sensitivity values are shown in Supplementary Figure 2A,C.

These cell types have frequently been reported to influence clinical response in BC, particularly in the context of chemotherapy-induced immunogenic cell death as the proposed mechanism [51][11]. Changes in the immune population, such as an increase in regulatory T-cells and myeloid-derived suppressor-like cells, and a decrease in CD8^+^ T-cells, have been associated with achieving pCR following NAC [6]. Myeloid cells have been implicated in therapy response through interactions with the malignant and endothelial cells, inducing chemoresistance *via* the *CXCL1/2* – *S100A8/9* loop, whereas tumor-associated macrophages have been linked to adverse outcomes and chemoresistance through survival factor secretion, anti-apoptotic pathway activation, and modulation of signaling pathways [6][15][16]. *VEGF*-mediated upregulation of survivin in Endothelial cells was further implicated in playing central roles in chemoresistance [15]. Additionally, plasmablasts, as precursors of plasma cells, have been associated with better prognosis [19][20], while B-cells have shown both pro- and anti-tumor roles [18][19][20].

We further tested and validated the predictive power of these cell types independently in external validation using the ARTemis + PBCP (*n* = 55) and BrighTNess (*n* = 106) cohorts, observing consistent results (Figure 2C–D, Supplementary Figure 2A,C). In the ARTemis + PBCP cohort (dominated by ER-positive, HER2-negative BC), the prominent contributors to chemotherapy response were CAFs, normal epithelial, and cancer epithelial (Figure 2C), whereas in the TNBC-specific BrighTNess cohort, the key players were myeloid, plasmablasts, and cancer epithelial (Figure 2D). When stratifying TransNEO and ARTemis + PBCP cohorts by the available BC subtypes, our cell-type-specific models retain good performance across both subtypes (Supplementary Figure 3A–D). The models for ER-positive, HER2-negative BC were more predictive than TNBC, possibly due to sample size limitations as well as increased heterogeneity in TNBC. These findings support the prominent involvement of immune and stromal cells alongside the malignant cells in mediating patient response to chemotherapy in the TME, with variations observed likely due to tumor subtype composition and ER/HER2 status differences across cohorts.

Our analysis did not identify T-cells as a prominent predictive cell type, as their expression provide marginal to no enhancement in predictive power over bulk expression (Figure 2A–B). This lack of signal may stem from aggregating different T-cell subtypes together (due to limits on the resolution of the deconvolution, which breaks down for low-abundance cell types). To study this further, we performed an enrichment analysis to delineate the contributions of CD4^+^ and CD8^+^ T-cells to chemotherapy response. However, the expression of each of these cell types still exhibited limited stratification power in distinguishing responders from non-responders across the three bulk cohorts (Supplementary Figure 4A–D). Additionally, we complemented the expression-based analysis with an analysis of the association between cell abundances and clinical response. This revealed that the overall T-cell abundance exhibited predictive power similar to or even higher than CD4^+^ and CD8^+^ subtypes (Supplementary Figure 4E–G), albeit still significantly lower than that of bulk transcriptomics.

### An ensemble model incorporating the expression of immune and stromal cells significantly boosts the predictive power of patient response

**Figure 3:**
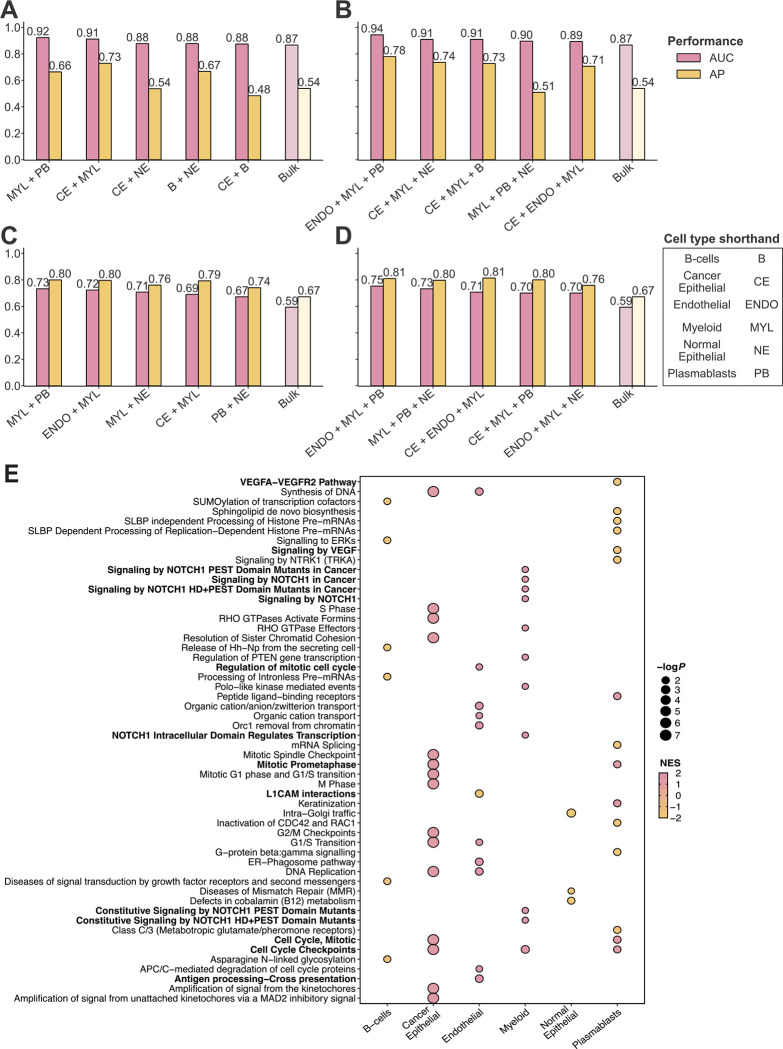
The prominent multi-cell-type ensembles mediating chemotherapy response in breast cancer tumor microenvironment. **A-B.** Validation performance with ARTemis + PBCP (*n* = 55) for the five most prominent two-cell-ensembles (**A**) and three-cell-ensembles (**B**) along with the bulk. AUC and AP stand for the area under the receiver operating characteristics curve and average precision (equivalent to the area under the precision-recall curve), respectively. Ensembles are ranked by their AUC values in a descending order. **C-D.** Validation performance with BrighTNess (*n* = 106) for the five most prominent two-cell-ensembles (**C**) and three-cell-ensembles (**D**) along with the bulk. Ensembles are ranked by their AUC values in a descending order. **E.** The enriched Reactome pathways (FDR-adjusted *p*-value ≤ 0.2) across the prominent cell types. For each cell type, only the top 10 relevant pathways are displayed. NES and log*P* stand for the normalized enrichment score from GSEA and log-scaled *p*-value, respectively.

**Figure 4:**
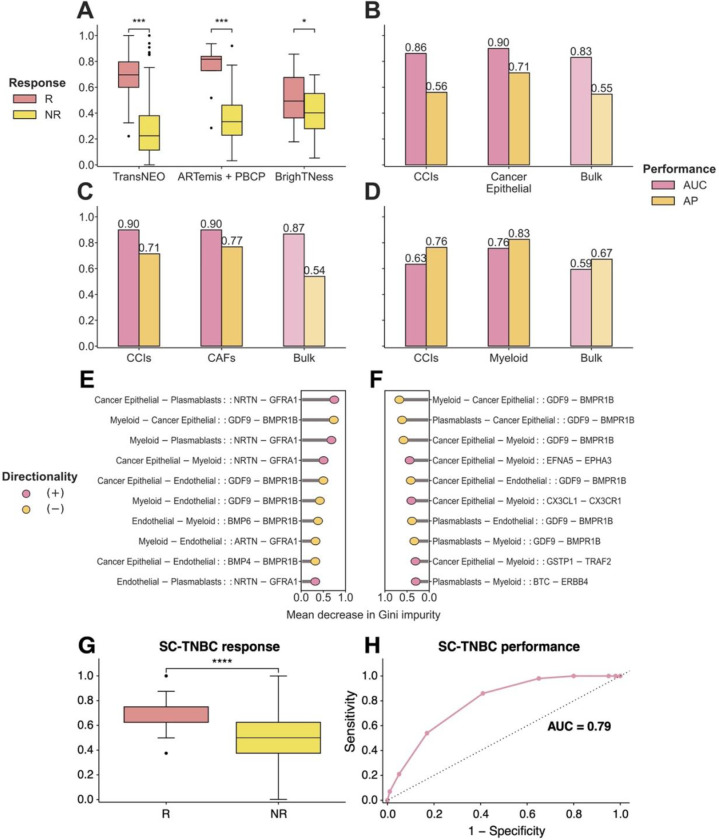
The prominent cell-cell interactions (CCIs) mediating chemotherapy response in breast cancer tumor microenvironment. **A.** Prediction scores for DECODEMi in cross-validation with TransNEO (*n* = 94) and in external validation with ARTemis + PBCP (*n* = 55) and BrighTNess (*n* = 106). R and NR stand for responders and non-responders, respectively. The differences between the prediction scores were computed by using a one-tailed Wilcoxon rank-sum test. **B.** Model performance in cross-validation with TransNEO for CCI-based, the top cell-type-specific, and bulk predictors. AUC and AP stand for the area under the receiver operating characteristics curve and average precision (equivalent to the area under precision-recall curve), respectively. **C-D.** Model performance in external validation with ARTemis + PBCP (**C**) and BrighTNess (**D**) for CCI-based, the top cell-type-specific, and bulk predictors. **E-F.** The 10 most predictive CCIs in external validation with ARTemis + PBCP (**E**) and BrighTNess (**F**). Each CCI is displayed as a quadruplet where the first pair containing the ligand and receptor cell types (separated by ‘−’) is separated by ‘::’ from the second pair containing the corresponding ligand and receptor genes (separated by ‘−’). Mean decrease in Gini impurity is the built-in feature importance measure in random forest where a higher value indicates a higher importance and *vice versa* [98]. Feature directionalities were computed using Fisher’s enrichment analysis. **G-H.** Prediction scores (**G**) and AUC value (**H**) for chemotherapy response prediction in external validation with the SC-TNBC cohort (*n* = 200, sourced from Zhang et al. [48]) using the top 170 response-relevant CCIs (among B-cells, myeloid, and T-cells) identified in bulk by DECODEMi.

To further enhance DECODEM’s performance, we aggregated multiple individual cell types together by appending the corresponding expression profiles as the input features. We explored 35 combinations of two to three cell types exhaustively, This comprehensive analysis yielded a boost in predictive performance in both ARTemis + PBCP and BrighTNess cohorts, with significant overlaps observed in the top multi-cell-type combinations across cohorts (Figure 3A–D, Supplementary Figure 2B,D). Remarkably, the ensemble of immune and stromal cells, specifically the combination of the expressions of endothelial, myeloid, and plasmablast cells exhibited the strongest stratification capability in identifying chemotherapy responders (*ARTemis + PBCP*: AUC = 0.94, AP = 0.78, Figure 3B; *BrighTNess*: AUC = 0.75, AP = 0.81, Figure 3D). Additionally, the immune-cell-ensemble comprising myeloid and plasmablasts displayed high stratification (*ARTemis + PBCP*: AUC = 0.92, AP = 0.66, Figure 3A; *BrighTNess*: AUC = 0.73, AP = 0.80, Figure 3C). These findings testify to the complementary contributions of different cell types within the TME to chemotherapy response. However, the top ensemble for the BrighTNess cohort provided a similar performance to the top cell type, myeloid (AUC = 0.76, AP = 0.83, Figure 2D), suggesting that the interactions among different cell types are less prominent in this cohort compared to the other cohorts, illustrated further in Figure 4D. The subtype-specific stratification of ARTemis + PBCP reinforced the superior predictive capabilities of these ensembles across both available BC subtypes while recapitulating their diminished impact in TNBC (Supplementary Figure 3E–F).

We conducted gene set enrichment analysis (GSEA) with Reactome pathways [52] to explore the functional contributions of these prominent cell types in chemotherapy response prediction. This analysis revealed the enrichment of cell cycle checkpoint and DNA replication pathways across different cell types (false discovery rate (FDR)-adjusted *p*-value ≤ 0.2; Figure 3E) as the most common functional contribution. Dysregulation of these pathways are frequent in BC and promotes tumor proliferation [53][54][55], making them valuable prognostic markers and therapeutic targets [56][57][58][59]. Immune cells exhibited enrichment in signal transduction pathways (*e.g.*, *VEGFA* – *VEGFR2* and *VEGF* signaling pathways in plasmablasts and multiple *NOTCH1* signaling pathways in myeloid), implicated in malignant progression and therapy resistance, particularly in TNBC [60][61][62][63][64][65][66]. Stromal endothelial cells showed enrichment in development- and immune-related pathways (*e.g.*, *L1CAM* interactions and antigen processing & presentation), contributing to aggressive BC progression and immune evasion with implications for both chemotherapy and immunotherapy [67][68][69][70][71][72][73].

To delve deeper into the underlying mechanism, we assessed DECODEM’s performance in light of the prevalent chemotherapy regimen within each cohort. TransNEO and ARTemis + PBCP predominantly employed T-FEC (*n* = 61 and 40, respectively; including docetaxel/taxotere, 5-flurouracil, epirubicin, and cyclophosphamide), while BrighTNess used a uniform regimen of paclitaxel and carboplatin (Supplementary Figure 1C). All six drugs have known clinical activities stimulating anticancer immunity *via* releasing immunostimulatory molecules from cancer cells (*on-target* effects) or promoting activation of effector cells and diminishing immunosuppressive cells (*off-target* effects) [73]. Remarkably, across all three cohorts spanning two BC subtypes and two regimens, DECODEM identified myeloid and plasmablasts to consistently stratify responders to *immunogenic* drug regimens, alongside malignant cells (Supplementary Figure 5A,B,D). For ARTemis + PBCP (dominated by ER-positive, HER2-negative BC), although the bulk mixture displayed comparable or superior predictive power than each of these two individual immune cells, their ensemble markedly boosted the performance, supporting the possibility that there may be prominent CCIs between these cell types that may be associated with the likelihood of response (Supplementary Figure 5C). Among the three-cell-ensemble models, the combination of endothelial, myeloid, and plasmablasts retains its highest performance, further underscoring the critical roles of the stromal – immune interactions. Additionally, for BrighTNess, the individual immune cell types showed notably strong predictive abilities, with less pronounced CCI effects (Supplementary Figure 5E, Figure 4D). Collectively, these findings corroborate the immunogenic effects mediated by chemotherapy, suggestive of *immunogenic cell death* [73] as suggested by Sammut et al. [11].

### Identifying key cell-cell interactions associated with chemotherapy response

To investigate the impact of the presence of interactions among multiple cell types in modulating chemotherapy response, we use DECODEMi to analyze cell-cell interactions (inferred using LIRICS [44], starting with 2,422 ligand – receptor (L-R) pairs from Ramilowski et al. [50]; see details in METHODS) that are predictive of patient response. As before, we conducted two analyses: (a) cross-validation on TransNEO (*n* = 94), and (b) external validation on ARTemis + PBCP (*n* = 55) and BrighTNess (*n* = 106). We found that CCIs encompassing only the four most prominent cell types in the TME (*cancer epithelial*, *endothelial*, *myeloid*, and *plasmablasts*) exhibited a comparable classification power to all available CCIs across all three cohorts (one-tailed Wilcoxon rank-sum test *p*-value ≤ 0.05), further testifying to the influential roles of these specific cell types in patient response (Figure 4A–D, Supplementary Figure 6A–D). The CCIs involving these four cell types markedly improved the predictive power of CCI-based predictors in both TransNEO and ARTemis + PBCP (Figure 4A–C). However, the performance of CCIs was generally lower than the top predictive myeloid cells in BrighTNess cohort (Figure 4D). This discrepancy can again be attributed to cohort composition and treatment-specific variations. It is worth noting that this cohort included only TNBC patients treated with paclitaxel and carboplatin, for which very few samples were available in training (Supplementary Figure 1B–C). These results reinforced that leveraging information from multiple cell types can notably improve clinical response prediction and the inter-cellular crosstalk may involve mediating treatment response in the TME. To further assess the generalizability of DECODEMi beyond bulk cohorts, we assessed its effectiveness on an independent single-cell (SC) TNBC cohort without additional training. To overcome the limitation of small sample size (n = 6), we constructed a SC-TNBC cohort of 200 ‘pseudopatients’ treated with chemotherapy (R:NR = 100:100) by downsampling 28,209 cells across the six TNBC patients who received paclitaxel in Zhang et al.’s study [48]. We focused on the CCIs among the three cell types (encompassing B-cells, myeloid, and T-cells) present in both SC and deconvolved bulk data, and identified the 170 top predictive CCIs (covering 134 L-R pairs) in bulk TransNEO cohort. The prediction scores for the SC-TNBC cohort were computed by counting the number of activated CCIs within each pseudopatient (as inferred by Kumar et al. [103]; see details in METHODS). Our analysis demonstrated the significant and robust predictive capabilities of these top CCIs (one-tailed Wilcoxon rank-sum test *p*-value ≤ 0.001, Figure 4G–H), affirming DECODEMi’s ability to identify the CCIs predictive of patient response.

By computing feature importance from DECODEMi, we identified the most predictive cell-to-cell communications across the ARTemis + PBCP and BrighTNess cohorts, finding a significant overlap in top CCIs (Figure 4E–F). Remarkably, the interaction between ‘*GDF9*’ (growth differentiation factor 9) and ‘*BMPR1B*’ (bone morphogenetic protein receptor 1B), both members of the TGF-β superfamily (the corresponding protein-protein interaction (PPI) network, obtained from STRING [100], is provided in Supplementary Figure 6E) with roles in reproductive biology and signal transduction [74][75][76][77], emerged as a key CCI with the same directionality across multiple cell types. These genes have been implicated in BC prognosis and therapeutic outcomes [78][79][80], with *BMPR1B* exhibiting dual roles in chemotherapy response [81][82][83]. Another top CCI involved the renowned *GDNF* – *RET* signaling pathway interactions between ‘*NRTN*’ (neurturin), or its paralog ‘*ARTN*’ (artemin), and ‘*GFRA1*’ (GDNF family receptor alpha 1) in multiple cell types (Figure 4E–F; see PPI network in Supplementary Figure 6F), which are all members of the glial cell line-derived neurotrophic factor (GDNF) family within the TGF-β superfamily with roles in neuronal survival, growth, and differentiation [84]. The GDNF family exhibits diverse roles in cancer [85][86], including an association with therapy resistance in ER-positive BC, highlighting *GFRA1* as a potential target [87][88][89][90]. Additionally, the well-studied chemokine interaction between ‘*CX3CL1*’ (C-X3-C chemokine ligand 1) and ‘*CX3CR1*’ (C-X3-C chemokine receptor 1) [91] is prominent in BrighTNess (Figure 4F; see PPI network in Supplementary Figure 6G), which is known to be involved in progression and metastasis across multiple cancers including BC, presenting a promising pan-cancer target [92][93][94][95][96][97].

Overall, DECODEMi application results in: (a) a simpler predictor leveraging CCIs that improves patient stratification compared to bulk transcriptomics, and (b) the identification of cell-to-cell communications predictive of treatment response, whose activation is associated with pro-cancerous roles, thus offering potential novel therapeutic targets.

### DECODEM identifies responders to chemotherapy and anti-PD-L1 therapy from single-cell transcriptomics

**Figure 5:**
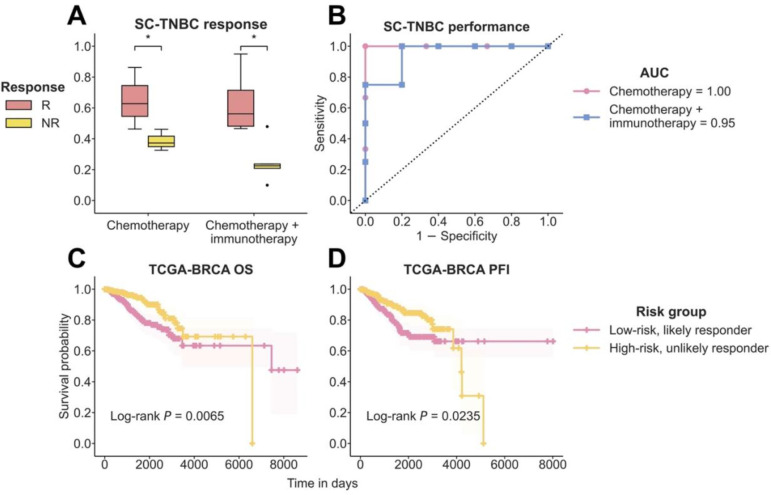
Generalizability of DECODEM to single-cell (SC) transcriptomics and for TCGA patient survival stratification. **A-B.** Prediction scores (**A**) and the area under the receiver operating characteristics curve (AUC) values (**B**) in external validation with SC expression of TNBC patients (SC-TNBC) treated with chemotherapy alone (*n* = 6) or combined with immunotherapy (*n* = 9) from Zhang et al. [48]. R and NR stand for responders and non-responders, respectively. The differences between the prediction scores were computed by using a one-tailed Wilcoxon rank-sum test. **C-D.** Kaplan-Meier plots depicting the overall survival (OS; **C**) and progression-free interval (PFI; **D**) of 705 BC patients from TCGA (TCGA-BRCA). Patients were stratified into low-risk, likely responder (*n* = 353) and high-risk, unlikely responder (*n* = 352) groups by their median DECODEM score computed using the expression of cancer epithelial cells, whereby the low-risk and high-risk groups comprised individuals with scores above and below the median, respectively. The differences between the two curves were computed by using a Log-rank test.

To further assess the generalizability of DECODEM, we evaluated the predictive capabilities of the cell-type-specific models with single-cell transcriptomics. We analyzed a SC cohort containing the expression of B-cells, myeloid, and T-cells for 15 patients with triple negative breast cancer from Zhang et al., who were either treated with chemotherapy (paclitaxel) alone or combined with the anti-PD-L1 agent, atezolizumab [48]. We first stratified the patient cohort into those who received chemotherapy alone (*n* = 6) and those who underwent chemotherapy + immunotherapy (*n* = 9). For each subset, we applied our established DECODEM models for these three cell types which were trained with deconvolved TransNEO data alongside a baseline ‘pseudobulk’ model (analogous to bulk in previous scenarios; see details in METHODS). Our findings revealed that the expression of B-cells exhibited the highest predictive potential within this cohort (Figure 5A–B, Supplementary Figure 7A–D). Notably, this B-cells-specific predictor from DECODEM effectively distinguishes the responders from non-responders for both intervention types (one-tailed Wilcoxon rank-sum test *p*-value ≤ 0.05, Figure 5A–B). These findings further attest to the generalizability and robustness of DECODEM, highlighting its potential in capturing treatment-invariant properties of the BC TME to predict response to the combination of chemotherapy and immunotherapy through SC transcriptomics.

### DECODEM effectively stratifies TCGA patients’ survival

We finally explored whether DECODEM can stratify the survival of 705 breast cancer patients in The Cancer Genome Atlas (TCGA-BRCA) from their deconvolved expression, obtained from Wang et al. [44]. Focusing on the expression of the prominent cell type of cancer epithelial (and endothelial) cells, DECODEM effectively stratified these patients into low-risk, likely responders (scores > median score, *n* = 353) and high-risk, unlikely responders (scores ≤ median score, *n* = 352), as displayed by their overall survival and progression-free interval (Log-rank test *p*-value ≤ 0.05, Figure 5C–D, Supplementary Figure 7E–F). Remarkably, this stratification was achieved regardless of treatment regimen, attesting that DECODEM captured the treatment-invariant properties of the BC TME that are predictive of patient survival.

## DISCUSSION

Our study introduces DECODEM, a transcriptomics-based modeling framework employing CODEFACS, a cutting-edge deconvolution tool, with a multi-stage machine learning pipeline to systematically assess the cell-type-specific contributions to predicting clinical response to treatment. As a representative scenario, we apply it to build predictors of patient response to neoadjuvant chemotherapy in breast cancer. We further present DECODEMi, a companion tool that identifies the key cell-cell interactions associated with clinical response. A recent study by Kester et al. investigated the association of cellular compositions of the BC TME with patient survival [43], but to our knowledge, the current study is the first-of-its-kind to analyze the cell-type-specific transcriptomics profiles in the BC TME to quantify their respective contributions to treatment response prediction.

We applied DECODEM to investigate the predictive power of the expression of various cell types in the BC TME to patient response to NAC. Our findings highlight the prominence of immune cells (*myeloid*, *plasmablasts*, *B-cells*) in chemotherapy response, consistent with Kester et al. [43], elucidating the complementary roles of diverse cell types that allows for the incorporation of top predictive cell types to build accurate clinical response predictors, and showcase DECODEMi’s ability to identify key CCIs impacting response across HER2-negative BC subtypes. Furthermore, we illustrated DECODEM’s generalizability to single-cell transcriptomics where it captures treatment-invariant properties of the TME, enabling accurate classification of responders for chemotherapy and immunotherapy, and its application for patient survival stratification in TCGA.

Immune cells have a clear association with prognosis in BC, both without chemotherapy and after chemotherapy in the early-stage setting [104][30][31]. The International TILs Working Group was established for the express purpose of standardizing incorporation of tumor infiltrating lymphocyte pathological review for clinical use, and established an online prognosis calculator for patients with TNBC (https://www.tilsinbreastcancer.org/prognosis-tool/). Plasma cells and naïve B-cells have been associated with better pCR rates in ER-positive tumors, whereas regulatory T-cells have been associated with better pCR rates in TNBC [105]. In our analysis, immune cells such as plasmablasts and myeloid cells exhibit involvement in signal transduction, particularly through *VEGF* signaling and *NOTCH* signaling pathways, which are associated with tumor-associated angiogenesis, enrichment of BC stem cells and epithelial-to-mesenchymal transition, leading to poor prognosis and therapy resistance [60][61][64][66]. Stromal cells such as endothelial cells show enrichments in development- and immune-related pathways including *L1CAM* interactions and antigen processing & presentation, with *L1CAM*’s oncogenic role and defects in antigen processing leading to poor outcomes and therapy resistance [67][68][69][70][71]. Overall, our results paired with the existing clinical evidence reinforce the notion that the presence of an active immune infiltration within the TME prior to treatment, as indicated by the prominent contributions of immune cells, leads to favorable response to chemotherapy, as suggested by Sammut et al. [11].

It is also important to note that DECODEM and DECODEMi offer to extend upon the existing application of gene expression in the early-stage breast cancer setting. Currently, gene expression-based tests such as Oncotype DX and MammaPrint are used for prediction and prognostication in ER-positive BC patients, reporting if a subset should receive chemotherapy due to worse predicted outcomes [106]. These tests use quantitative RT-PCR or cDNA microarray to assess a known subset of genes, and largely apply only to a subset of early-stage ER-positive BC patients. DECODEM and DECODEMi provide complimentary context to these tools, expanding the applicable subtypes for chemotherapy response prediction as well as greater biological context.

Although we presented a comprehensive framework to assess the TME in a quantitative manner, certain limitations should be acknowledged and addressed in future studies. *First*, our predictors rely on data-driven algorithms involving deconvolution and ML, necessitating a large sample size to attain statistical robustness and avoid overfitting. Accordingly, the available datasets are limited in size, limiting our ability to perform robust subtype-specific analyses. *Second*, any limitations inherent to CODEFACS, such as discrepancies in RNA-seq processing and reliance on appropriate molecular signatures [44], will affect DECODEM in the downstream analysis. The same goes for DECODEMi and its dependence on LIRICS using an appropriate ligand – receptor database [43]. *Third*, our deconvolution analysis could not be performed separately for the CD4^+^ and CD8^+^ T-cell subsets. Alternatively, we provided an enrichment analysis assessing the predictive powers of CD4^+^ and CD8^+^ T-cells which corroborated our findings from using the overall T-cells.

Our work focused on examining the association of the BC TME, specifically in HER2-negative subtypes, to clinical response to NAC. However, both DECODEM and DECODEMi can be applied to investigate diverse treatment regimens in different cancer indications. Although both TransNEO and ARTemis + PBCP cohorts include different chemotherapy combinations, limited sample size prevented us from exploring further. Therefore, an intriguing direction could be to investigate the distinct role of the TME in different chemotherapy families with different mechanisms of actions (MOAs) *e.g.*, taxanes, antimetabolites, platinum agents and so on. With DECODEMi, one could further pinpoint the family-specific significant cell-cell interactions to understand the underlying MOA and uncover novel CCIs with promising therapeutic implications. Additionally, expanding the investigation beyond the scope of cancer and examining the impact of the microenvironment in various indications such as aging-related disorders and autoimmune diseases could provide further valuable insights.

In summary, our study introduces two data-driven computational frameworks that enable systematic investigation of the roles of the microenvironment in various processes, including treatment response. These frameworks can utilize bulk transcriptomics from healthy and diseased conditions when single-cell information is not easily accessible, providing a valuable approach to study the TME in a comprehensive manner. Their application in breast cancer uncovers the considerable predictive power of diverse cell types within the TME and highlights key stromal – immune cell-cell interactions that are strongly predictive of patient response.

## METHODS

### Overview of the datasets

We analyzed three bulk transcriptomics datasets, encompassing a total of 255 breast cancer patients who received neoadjuvant chemotherapy (Supplementary Figure 1). The first dataset, recently published by Sammut et al., was the TransNEO cohort which included 94 HER2-negative breast tumors patients undergoing various NAC regimens [11]. Notably, Sammut et al. presented the complete TransNEO cohort, covering 168 patients with early and locally advanced BC from all major subtypes, and providing DNA-seq, RNA-seq, digital pathology, and treatment information for various subsets of patients [11]. The second dataset was the ARTemis + PBCP cohort, validated externally by Sammut et al., combining the control arm of the ARTemis trial (*n* = 38) received the combination therapy of FEC-T [46] and the HER2-negative cases from the Personalised Breast Cancer Programme (PBCP; *n* = 17) undergoing various NAC regimens [11] (Supplementary Figure 1B–C). The third dataset, the BrighTNess cohort, included 106 patients from arm B of the BrighTNess trial, a randomized, double-blind, placebo-controlled three-arm clinical study for veliparib in stage II/III TNBC [47]. Arm B included only patients who received the NAC combination of paclitaxel, carboplatin, and a placebo for veliparib (Supplementary Figure 1B–C). In all three cohorts, patient response was assessed at surgery using the residual cancer burden (RCB) index where patients achieving pathological complete response (pCR; RCB = 0) were considered as responders (R) and patients with residual diseases (RCB-I, II, III) as non-responders (NR) with different R:NR ratios (Supplementary Figure 1A).

Furthermore, we analyzed a recent single-cell cohort presented by Zhang et al. comprising 22 patients with advanced triple negative breast cancer. Half of the patients received paclitaxel alone, while the other half received paclitaxel combined with the anti-PD-L1 agent, atezolizumab [48]. This study included 78 tumor biopsies and blood samples collected at three time-points, including baseline. Zhang et al. provided the single-cell expression for four immune cell types (B-cells, T-cells, myeloid, and innate lymphoid cells) and patient response assessed using the Response Evaluation Criteria in Solid Tumors (RECIST) criteria [48]. We categorized the patients achieving complete or partial response as responders (R) and those with stable or progressive diseases as non-responders (NR). Our analysis focused solely on pre-treatment tissue samples, resulting in two treatment groups: (a) chemotherapy (*n* = 6, R:NR = 3:3) and (b) chemotherapy + immunotherapy (*n* = 9, R:NR = 4:5). To align with the output of CODEFACS, which provides the mean cell-type-specific expression profile per patient [44], we harmonized the cell-type-specific SC expression by averaging across all corresponding cells for each patient, while filtering for low-variance genes (threshold = 0.8). Additionally, we calculated the ‘pseudobulk’ expression as the mean expression across all cells encompassing the three cell types for each patient (post low-variance filtering) to generate a baseline expression profile similar to the bulk mixture.

### Deconvolution with CODEFACS

The first step of DECODEM involves employing CODEFACS, a cellular deconvolution tool recently developed in our lab [44]. CODEFACS characterizes the TME by reconstructing the cell-type-specific expression in each sample from the input bulk expression, utilizing the corresponding cell type abundance or a cell-type-specific signature profile as another input. The output includes the cell-type-specific expression profiles, the corresponding cell type abundances, and a confidence score matrix that quantifies the confidence level (as a value between 0 and 1) of the estimated expression values for each gene in each cell type across all samples.

We derived a cell type signature matrix of 1,400 genes representing nine cell types from a single-cell RNA-seq (scRNA-seq) cohort from Wu et al. [45] using an in-house signature derivation tool based on the CIBERSORTx algorithm [101]. We then deconvolved the three bulk expression profiles with this signature into cell-type-specific expression profiles, encompassing the nine cell types: *B-cells*, *Cancer-associated Fibroblasts (CAFs)*, *Cancer Epithelial (malignant)*, *Endothelial*, *Myeloid*, *Normal Epithelial*, *Plasmablasts*, *Perivascular-like Cells (PVL)*, and *T-cells*.

### Extraction of cell-cell interactions with LIRICS

We extended DECODEM to DECODEMi by incorporating multi-cell-type information through cell-cell interactions. To this end, DECODEMi employs LIRICS [44] on the output of CODEFACS to infer the activity of the corresponding CCIs. LIRICS applies a two-step approach to infer the cell-type-specific ligand – receptor (L-R) pairs that are likely to be ‘active’ in each sample from deconvolved expression. The first step is to query a database of 49,397 plausible L-R interactions between each pair of cell types in BC TME (see below for details). The second step infers an L-R interaction to be active (indicated by ‘1’) in a pair of cell types in a given sample if the expressions of both ligand and receptor genes exceed their median cell-type-specific expression values or inactive (indicated by ‘0’) otherwise. We used this binary CCI activity matrix output from LIRICS to assess the predictive potential of cell-to-cell communications.

### Curation of cell-type-specific ligand – receptor database for BC TME from single-cell transcriptomics

To infer the cell-cell interaction activity using LIRICS, we applied a two-step approach to construct a database of cell-type-specific ligand – receptor interactions (LRIs) to characterize all cell-to-cell communications that are likely to occur in the TME of BC patients. We first obtained a list of 2,422 potential L-R pairs, as annotated by Ramilowski et al. [50]. We next evaluated the potential of the L-R pairs occurring between the nine cell-types-of-interest using scRNA-seq data from Wu et al [45]. To discern the activity of the curated L-R list, we pooled together all 130,246 cells for the nine cell types from Wu et al. and adopted an empirical *p*-value based CCI inference approach following Kumar et al. [103], where an LRI with *p*-value ≤ 0.05 is categorized as ‘likely’ to occur in the TME of BC patients and ‘unlikely’ otherwise. We constructed a database of 49,397 cell-type-specific L-R pairs (*i.e.*, CCI quadruplets) that are likely to occur in BC TME. This database composed the initial feature space for LIRICS to yield different numbers of CCIs across three bulk cohorts: TransNEO = 46,755, ARTemis + PBCP = 43,887, and BrighTNess = 38,285.

### Machine learning pipeline with deconvolved expression and CCIs

In both DECODEM and DECODEMi, the second step involves building ML predictors of clinical response using cell-type-specific expression profiles and CCIs, respectively. For DECODEM, we built nine cell-type-specific predictors to classify responders and non-responders following treatment using a four-stage ML pipeline (Figure 1): (a) prefiltering to remove genes with CODEFACS confidence score < 0.99, (b) low-variance filtering to remove genes with variance < 0.1, (c) feature selection to select the top ‘*m*’ genes maximizing performance, where 2 ≤ *m* ≤ 25 with genes ranked by their ANOVA F-scores for association with response followed by standardization to scale each gene to zero mean and unit variance, and (d) classification with an unweighted ensemble classifier comprised of regularized logistic regression, random forest, support vector machine, and XGBoost (extreme gradient boosting) to predict the probabilities of achieving pCR. Here, the upper limit on the feature size is chosen to avoid model overfitting and longer runtimes (and evaluated to provide similar performance across different feature sizes in cross-validation). Furthermore, each prediction vector was rescaled between 0 and 1 to improve generalization for external validation. We trained this ML pipeline with three-fold cross-validation (CV) for hyperparameter tuning to maximize the AUC for each classifier separately. To avoid overfitting, we repeated the training procedure five times with five different CV splits and took the mean of the five prediction vectors as our final prediction. We analyzed the three bulk expression cohorts with this pipeline in two scenarios: (a) cross-validation with TransNEO (*n* = 94) where we applied an additional five-fold CV to estimate the performance, and (b) external validation on ARTemis + PBCP (*n* = 55) and BrighTNess (*n* = 106) using predictors trained on TransNEO. We further built a baseline predictor using bulk expression in ML pipeline directly and identified the ‘prominent’ cell types in CV that outperformed the bulk, subsequently validated in external validation.

To accommodate the sparse nature of SC transcriptomics for external validation with Zhang et al. [48], we slightly modified the pipeline to enable modeling for all available genes post filtering in step (c) *i.e.*, 2 ≤ *m* ≤ ‘all’. Additionally, for CCI-based analysis with DECODEMi, we further updated the low-variance cut-off in step (b) to 0.08 and the classifier in step (d) to a random forest that is better suited for the binary nature of the CCI matrix and includes a built-in measure of feature importance, mean decrease in Gini impurity (similar performance to the permutation-based approaches) [98]. The corresponding CCI directionalities were computed using a two-tailed Fisher’s test (followed by false discovery rate (FDR) adjustment for multiple hypothesis correction *i.e.*, FDR-adjusted *p*-value ≤ 0.05), where an odds ratio (OR) > 1 denotes an enrichment in responders and OR < 1 indicates enrichment in non-responders. Moreover, to filter CCIs for a subset of cell types, we only include the interactions involving the confident L-R genes (CODEFACS confidence level ≥ 0.99) in the corresponding cell type.

### CD4^+^ / CD8^+^ T-cells enrichment analysis

To analyze the contribution of the individual T-cell subtypes to chemotherapy response, we performed a gene set variation analysis (GSVA) to summarize pathway activity per sample for each T-cell subtype. We employed two single-cell expression signatures derived from Wu et al. [45], collected *via* the Tumor Immune Single Cell Hub 2 (TISCH2) [102]. These signatures consisted of 207 and 424 differentially expressed genes (log-fold-change ≥ 0.5) for CD4^+^ and CD8^+^ T-cells, respectively (with an overlap of 151 genes). Subsequently, we evaluated the association of the GSVA enrichment scores (rescaled between 0 and 1) with clinical response across the three bulk cohorts through a one-tailed Wilcoxon rank-sum test (FDR-adjusted *p*-value ≤ 0.05), and also assessed the AUC and AP values independently.

### Cell-type-specific enrichment analysis

We conducted gene set enrichment analysis (GSEA) to investigate the association of each cell type with different biological processes. For each cell type, we first ranked the 17,680 protein-coding genes available in TransNEO by their association with positive outcome (*i.e.*, pCR), measured by the *p*-values from a one-tailed Wilcoxon rank-sum test, adjusted by CODEFACS confidence scores. We next performed a separate GSEA analysis using the Reactome pathways [52] for each prominent cell type (except CAFs). This resulted in six different sets of enriched pathways (FDR-adjusted *p*-value ≤ 0.2) across six prominent cell types. From these sets, we reported the top 10 relevant pathways for each cell type.

### Single-cell validation of top cell-cell interactions

We validated the most predictive cell-cell interactions from DECODEMi using a single-cell cohort from Zhang et al. that includes six TNBC patients treated with chemotherapy [48]. We devised a three-step validation procedure: *First,* for consistency, we retrained DECODEMi using only the interactions among cell types available in both bulk and SC (namely *B-cells*, *myeloid*, and *T-cells*) in TransNEO. This resulted in the extraction of the top predictive CCIs (*m* = 170) involving a total of 134 unique L-R pairs. *Second*, to enhance the statistical power, we pooled the 28,209 cells encompassing these three cell types from the six chemotherapy patients in Zhang et al., and randomly downsampled 30% of the cells 100 times within each treatment group for each cell type. This procedure generated a profile of 200 ‘pseudopatients’ (R:NR = 100:100) with empirical *p*-values encompassing the significance levels of the 804 possible CCIs involving the 134 L-R pairs in three cell types (= 134 × 6) *i.e.*, the ‘SC-TNBC cohort’. To identify the activation status of the 170 CCIs-of-interest in this cohort, we again adopted the CCI inference approach from Kumar et al. [103], where a CCI is denoted as ‘active’ if *p*-value ≤ 0.05 and ‘inactive’ otherwise. *Third*, for each pseudopatient, we predicted the chemotherapy response as the sum of the active CCIs, weighted by their directionalities (*i.e.*, +1 or −1 when OR > 1 or < 1, FDR-adjusted *p*-value ≤ 0.05; and 0 otherwise), followed by a rescaling between 0 and 1 across all pesudopatients. We used these prediction scores to evaluate the predictive performance of these top CCIs learned from bulk on the SC-TNBC cohort through using a one-tailed Wilcoxon rank-sum test (FDR-adjusted *p*-value ≤ 0.05) and receiver operating characteristics (ROC) analysis.

## Supplementary Material

Supplement 1

## Data Availability

For TransNEO cohort, RNA-seq counts and clinical data were acquired from Sammut et al. (https://doi.org/10.1038/s41586-021-04278-5). RNA-seq counts were converted to TPM by using Ensembl gene annotations (GRCh37.87, http://ftp.ensembl.org/pub/grch37/release-87/gtf/homo_sapiens/). For ARTemis + PBCP cohort, clinical data were collected from Sammut et al. (https://doi.org/10.1038/s41586-021-04278-5) and RNA-seq TPM values were obtained directly from the authors. For BrighTNess cohort, RNA-seq FPKM values and clinical data were acquired from GEO (https://www.ncbi.nlm.nih.gov/geo/query/acc.cgi?acc=GSE164458). The FPKM values were converted into TPM. For Zhang et al. cohort, scRNA-seq counts and clinical data were obtained from Zhang et al. (https://doi.org/10.1016/j.ccell.2021.09.010). For the single-cell signature for deconvolution, scRNA-seq counts and cell annotations were collected from Wu et al. (https://doi.org/10.1038/s41588-021-00911-1). The ligand-receptor list for LIRICS were obtained from Ramilowski et al. via GitHub (https://github.com/LewisLabUCSD/Ligand-Receptor-Pairs). The deconvolved expression and cell fraction data using CODEFACS on TCGA-BRCA patients were collected from Wang et al. (https://doi.org/10.1158%2F2159-8290.CD-21-0887). The corresponding TCGA-BRCA patients’ overall survival and progression-free interval (synonymous to progression-free survival) data were obtained from the UCSC Xena browser (https://xenabrowser.net). All data used in this study are either publicly available or available upon request to the original authors publishing the data. All deconvolved expression data generated in this study will be made publicly available upon publication.
