# Supplementary material for: Enhanced prediction of breast cancer patient response to chemotherapy by integrating deconvolved expression patterns of immune, stromal and tumor cells
Source: bioRxiv. 2025 Jul 10:2024.06.14.598770. Originally published 2024 Jun 14. Preprint. [Version 2] doi: 10.1101/2024.06.14.598770 (PMC11451622; doi:10.1101/2024.06.14.598770)
Supplement: Supplement 1 [file NIHPP2024.06.14.598770v2-supplement-1.pdf]

bioRxiv preprint doi: https://doi.org/10.1101/2024.06.14.598770; this version posted July 10, 2025. The copyright holder for this preprint (which was not certified by peer review) is the author/funder. This article is a US Government work. It is not subject to copyright under 17 USC 105 and is also made available for use under a CC0 license.

SUPPLEMENTARY FIGURES

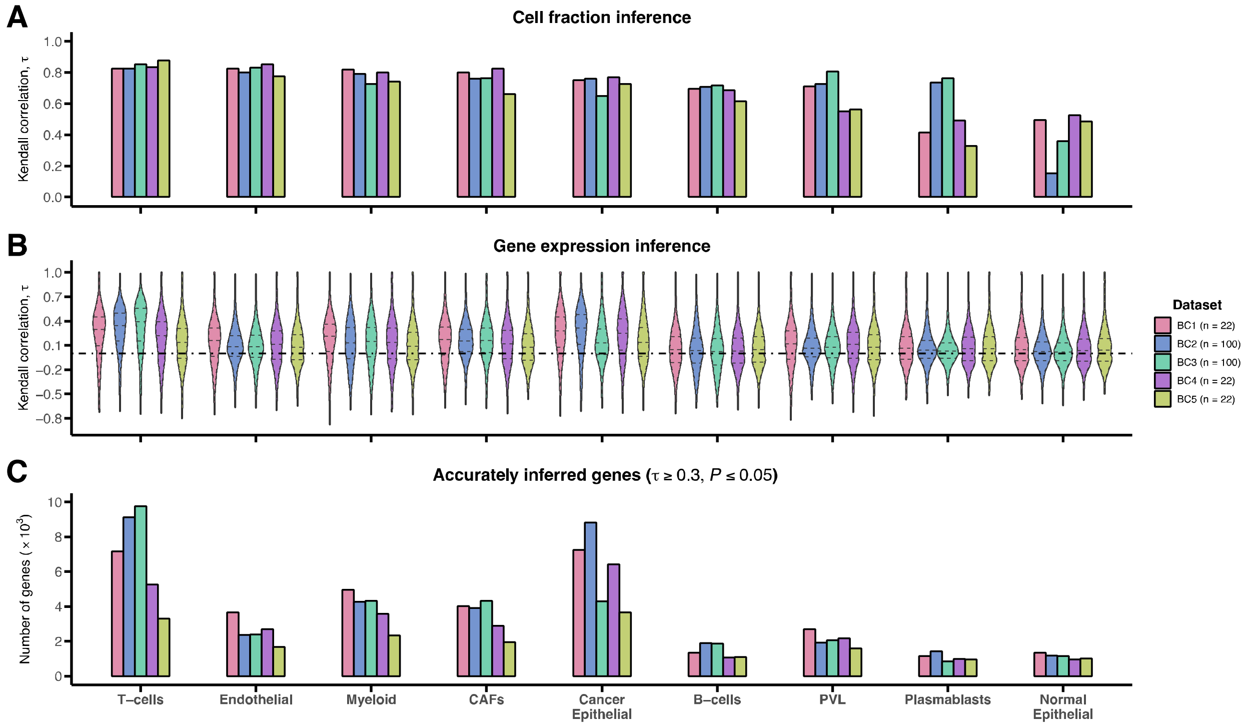

Supplementary Figure 1: Benchmarking CODEFACS on five breast cancer cohorts generated from single-cell (SC) data.

A. Correlation of actual and CODEFACS-inferred cell fraction values for nine cell types across five benchmark cohorts (BC1-5), generated *via* mixing SC expression from Wu et al. (see **METHODS**). Cell types are ranked by their mean correlation across the five cohorts in a descending order.

B. Correlation of actual and CODEFACS-inferred cell-type-specific gene expression values for nine cell types across five benchmark cohorts. Cell types are ranked by their mean correlation for cell abundance accuracy in **A**.

C. Number of accurately inferred genes for nine cell types across five benchmark cohorts. Cell types are ranked by their mean correlation for cell abundance accuracy in **A**.

bioRxiv preprint doi: https://doi.org/10.1101/2024.06.14.598770; this version posted July 10, 2025. The copyright holder for this preprint (which was not certified by peer review) is the author/funder. This article is a US Government work. It is not subject to copyright under 17 USC 105 and is also made available for use under a CC0 license.

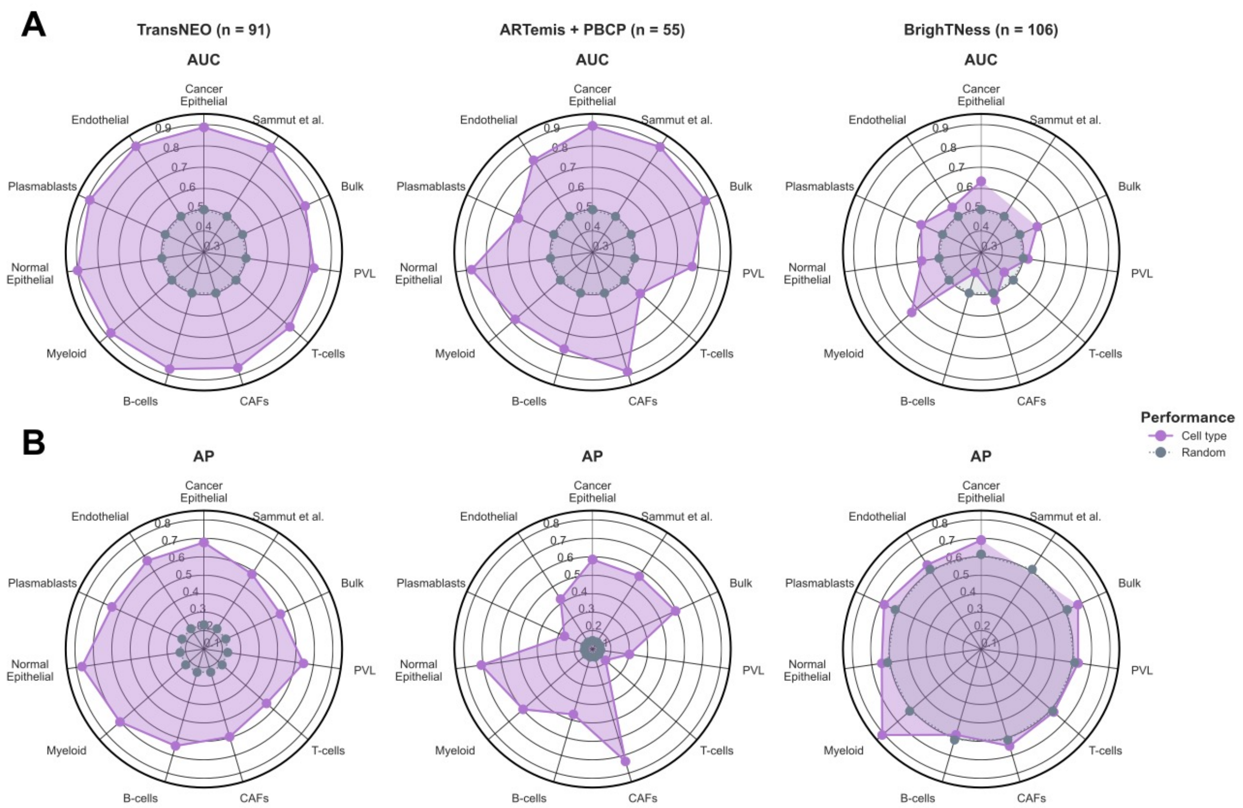

Supplementary Figure 2: Hyperparameter tuning with leave-one-out cross-validation (CV) leads to similar cell-type-specific performance to using three-fold cross-validation.

A-B. Comparison of model performance for nine cell-type-specific, bulk and Sammut et al. (for first two cohorts) predictors across TransNEO, ARTemis + PBCP and BrighTNess, where model hyperparameters were optimized using leave-one-out CV. AUC and AP stand for the area under the receiver operating characteristics curve and average precision, respectively. ‘Random’ denotes a random predictor (AUC = 0.5, AP = fraction of responders). Cell types are ranked by their original AUC values for TransNEO (obtained using three-fold CV for hyperparameter tuning; see **Figure 2A**) in the counterclockwise direction.

bioRxiv preprint doi: https://doi.org/10.1101/2024.06.14.598770; this version posted July 10, 2025. The copyright holder for this preprint (which was not certified by peer review) is the author/funder. This article is a US Government work. It is not subject to copyright under 17 USC 105 and is also made available for use under a CC0 license.

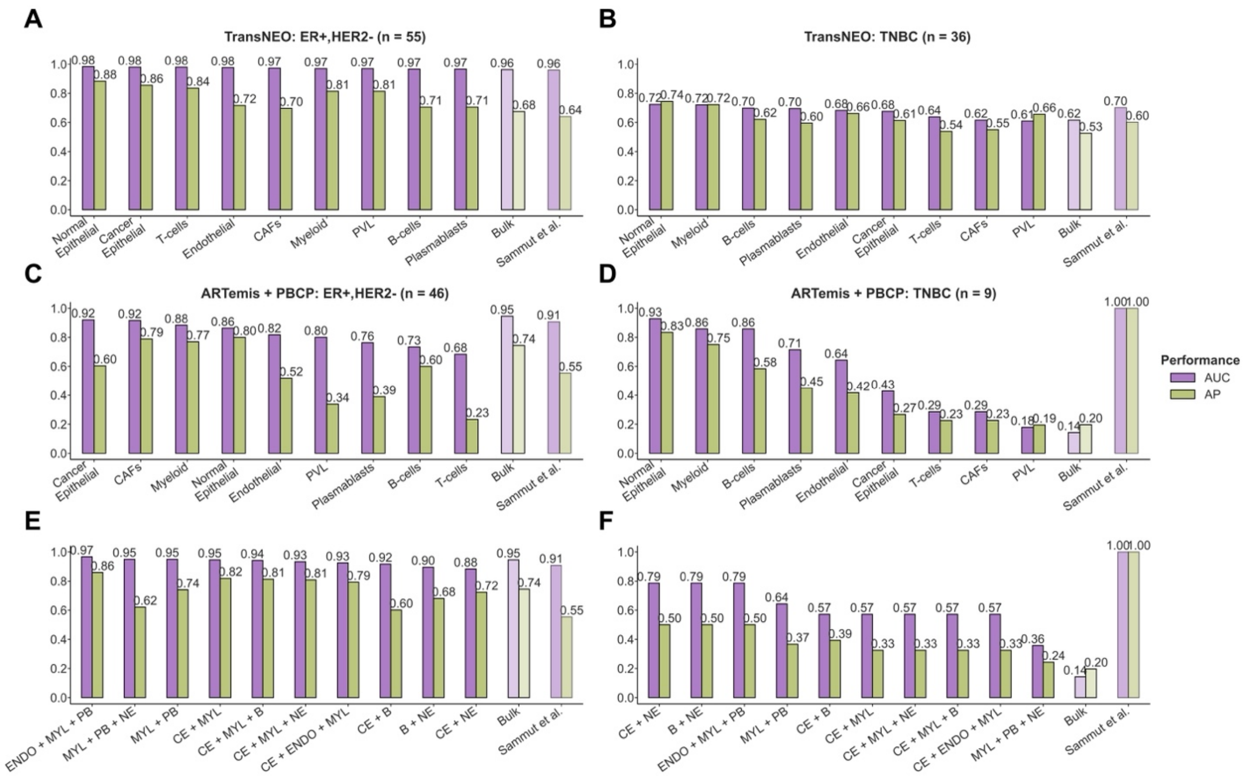

Supplementary Figure 3: The prominent cell types and ensembles mediating chemotherapy response in breast cancer tumor microenvironment in a subtype-specific manner.

A-B. Comparison of model performance across TransNEO for the ER+,HER2- (**A**) and TNBC (**B**) subtypes for nine cell-type-specific, bulk and Sammut et al. predictors. AUC and AP stand for the area under the receiver operating characteristics curve and average precision (equivalent to the area under the precision-recall curve), respectively. Cell types are ranked by their AUC values in a descending order.

C-D. Comparison of model performance across ARTemis + PBCP for the ER+,HER2- (**C**) and TNBC (**D**) subtypes for nine cell-type-specific, bulk and Sammut et al. predictors. Cell types are ranked by their AUC values in a descending order.

E-F. Comparison of model performance across with ARTemis + PBCP for the ER+,HER2- (**E**) and TNBC (**F**) subtypes for the five most prominent two-cell-ensembles, five most prominent three-cell-ensembles, bulk and Sammut et al. predictors. Ensembles are ranked by their AUC values in a descending order.

bioRxiv preprint doi: https://doi.org/10.1101/2024.06.14.598770; this version posted July 10, 2025. The copyright holder for this preprint (which was not certified by peer review) is the author/funder. This article is a US Government work. It is not subject to copyright under 17 USC 105 and is also made available for use under a CC0 license.

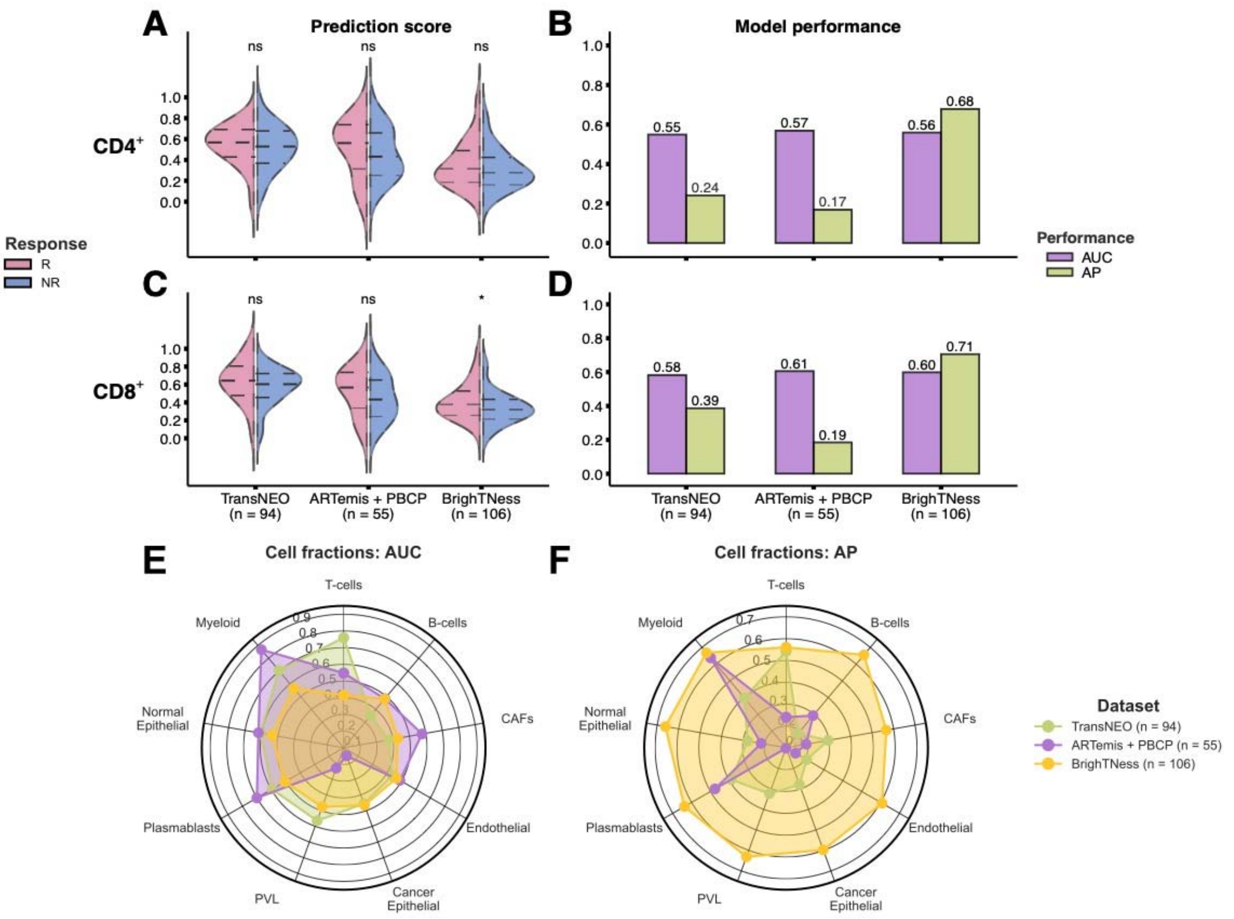

Supplementary Figure 4: The CD4+ / CD8+ T-cells enrichment and cell abundance – clinical response association analyses reaffirm the poor chemotherapy response stratification by T-cells at the current resolution.

A-B. GSVA enrichment scores (**A**) and predictive performance (**B**) of CD4+ T-cells for chemotherapy response stratification across TransNEO, ARTemis + PBCP and BrighTNess. R and NR stand for responders and non-responders, respectively. The differences between the prediction scores were computed by using a one-tailed Wilcoxon rank-sum test (* and 'ns' denote $P \leq 0.05$ and $P > 0.05$, respectively). AUC and AP stand for the area under the receiver operating characteristics curve and average precision (equivalent to the area under precision-recall curve), respectively.

C-D. GSVA enrichment scores (**C**) and predictive performance (**D**) of CD8+ T-cells for chemotherapy response stratification across the three cohorts.

bioRxiv preprint doi: https://doi.org/10.1101/2024.06.14.598770; this version posted July 10, 2025. The copyright holder for this preprint (which was not certified by peer review) is the author/funder. This article is a US Government work. It is not subject to copyright under 17 USC 105 and is also made available for use under a CC0 license.

E-F. Predictive performance of abundances of the nine cell types across the three cohorts. Cell types are ranked by their AUC values for TransNEO in a descending order in the counterclockwise direction.

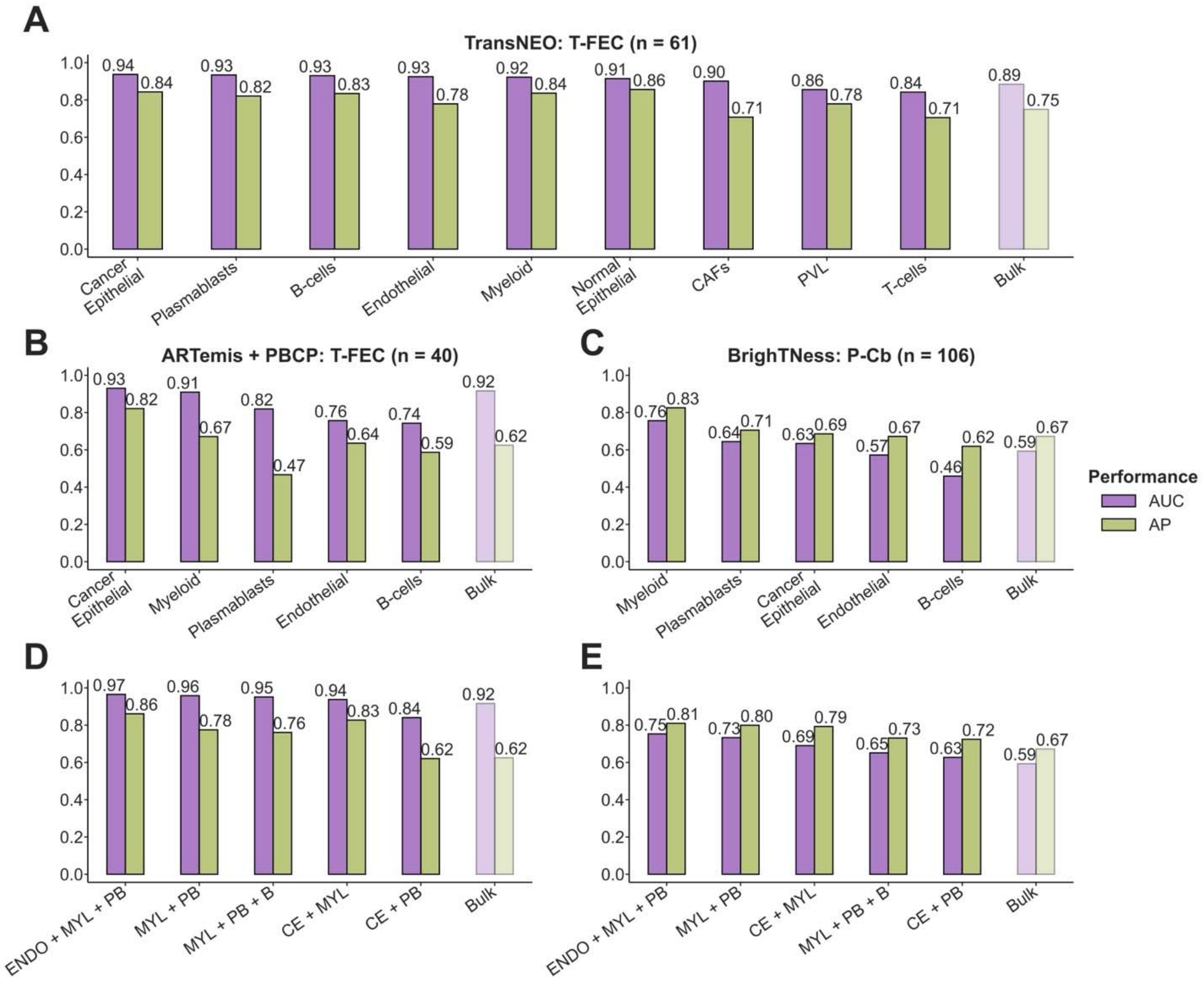

Supplementary Figure 5: The prominent immune cell types and ensembles mediating response to the most prevalent immunogenic chemotherapy regimen in each breast cancer cohort.

A. Comparison of model performance for nine cell-type-specific and bulk predictors across TransNEO for the T-FEC regimen. AUC and AP stand for the area under the receiver operating characteristics curve and average precision (equivalent to the area under the precision-recall curve), respectively. *C*, *E*, *F* and *T* stand for cyclophosphamide, epirubicin, 5-flurouracil and

bioRxiv preprint doi: https://doi.org/10.1101/2024.06.14.598770; this version posted July 10, 2025. The copyright holder for this preprint (which was not certified by peer review) is the author/funder. This article is a US Government work. It is not subject to copyright under 17 USC 105 and is also made available for use under a CC0 license.

docetaxel (taxotere), respectively. Cell types are ranked by their AUC values in a descending order.

B-C. Comparison of model performance for the top five cell-type-specific and bulk predictors across ARTemis + PBCP for the T-FEC regiment (**B**) and BrighTNess for the paclitaxel – carboplatin (P-Cb) regimen (**C**). Cell types are ranked by their AUC values in a descending order.

D-E. Comparison of model performance for the top five multi-cell-type ensemble and bulk predictors across ARTemis + PBCP for the T-FEC regiment (**D**) and BrighTNess for the P-Cb regimen (**E**). Ensembles are ranked by their AUC values in a descending order.

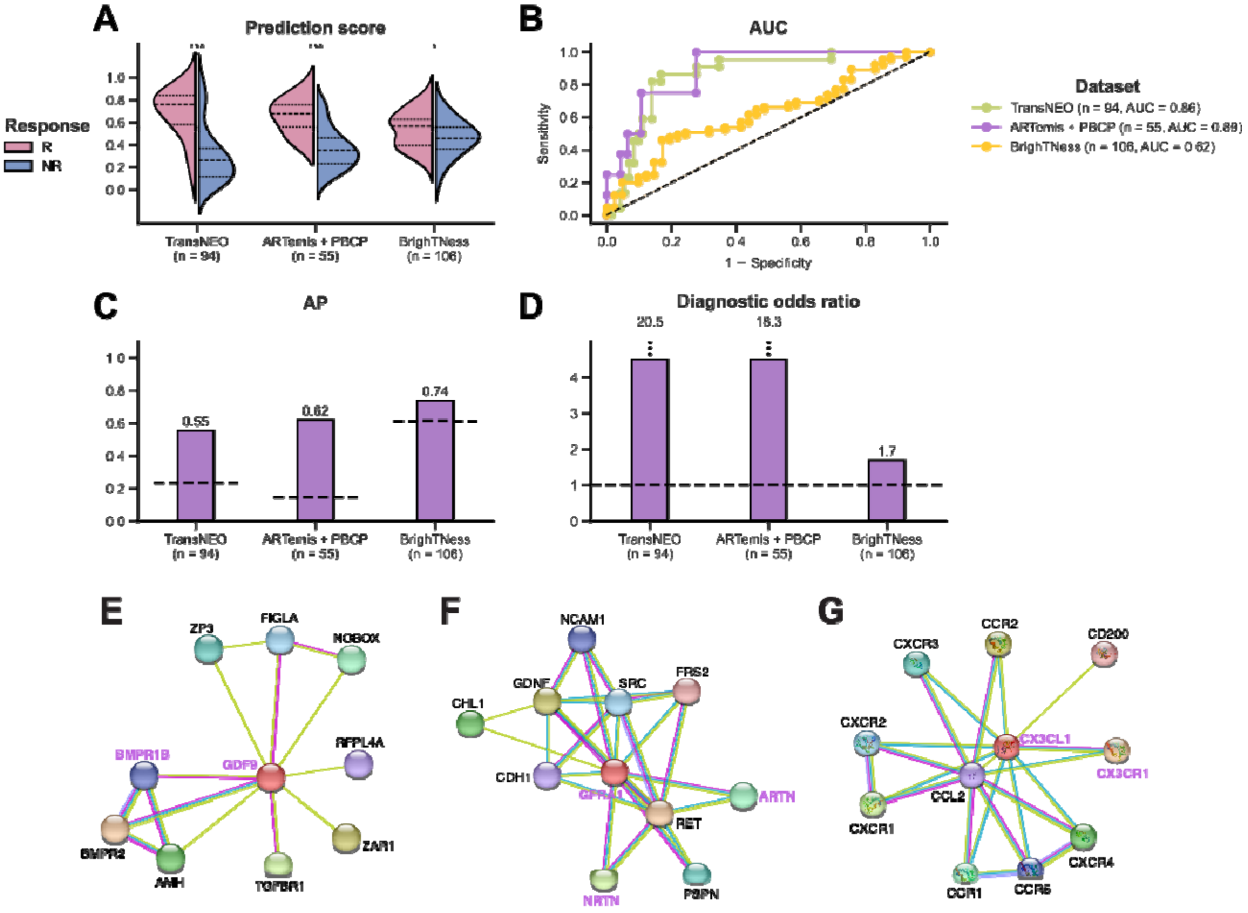

Supplementary Figure 6: Cell-cell interactions (CCIs) encompassing all cell types in mediating chemotherapy response in breast cancer tumor microenvironment.

A-D. Comparison of prediction scores (**A**) and model performance (**B-D**) for DECODEMi CCI-based predictor using all available CCIs across TransNEO, ARTemis + PBCP and BrighTNess. R and

bioRxiv preprint doi: https://doi.org/10.1101/2024.06.14.598770; this version posted July 10, 2025. The copyright holder for this preprint (which was not certified by peer review) is the author/funder. This article is a US Government work. It is not subject to copyright under 17 USC 105 and is also made available for use under a CC0 license.

NR stand for responders and non-responders, respectively. The differences between the prediction scores were computed by using a one-tailed Wilcoxon rank-sum test (*** and * denote $P \leq 0.001$ and $P \leq 0.05$, respectively). AUC and AP stand for the area under the receiver operating characteristics curve and average precision, respectively. The dotted lines in **C-D** represent the AP and diagnostic odds ratio (DOR) values for a random predictor (AP = fraction of responders, DOR = 1.0).

E-G. Protein-protein interaction networks obtained from STRING [76] displaying the known interaction networks for three prominent CCIs *i.e.*, *GDF9* – *BMPR1B* (**E**), *NRTN* – *GFRA1* and *ARTN* – *GFRA1* (**F**) and *CX3CL1* – *CX3CR1* (**G**).

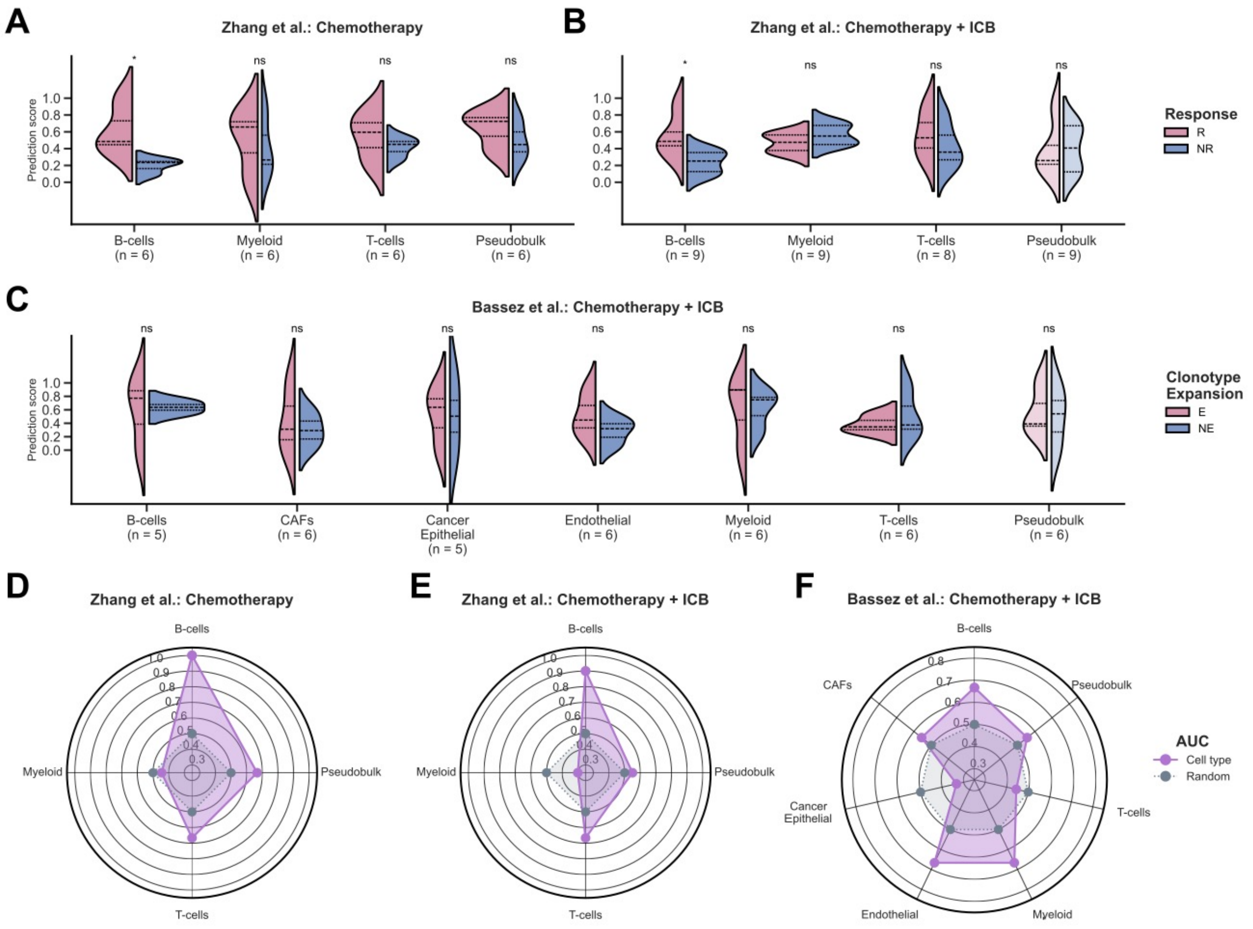

Supplementary Figure 7: DECODEM generalizes to single-cell (SC) transcriptomics for patient response prediction.

bioRxiv preprint doi: https://doi.org/10.1101/2024.06.14.598770; this version posted July 10, 2025. The copyright holder for this preprint (which was not certified by peer review) is the author/funder. This article is a US Government work. It is not subject to copyright under 17 USC 105 and is also made available for use under a CC0 license.

A-B. Comparison of prediction scores for the cell-type-specific and pseudobulk (similar to bulk in previous scenarios) predictors across SC expression for predicting response to neoadjuvant chemotherapy (NAC) alone (**A**) or in combination with immune checkpoint blockade (ICB) therapy (**B**) from Zhang et al., or predicting T-cell clonotype expansion following treatment with NAC and ICB therapy (**C**) from Bassez et al. R, NR, E and NE stand for responders, non-responders, expanders and non-expanders, respectively. The differences between the prediction scores were computed by using a one-tailed Wilcoxon rank-sum test (* and 'ns' denote $P \leq 0.05$ and $P > 0.05$, respectively).

D-F. Comparison of model performance for the cell-type-specific and pseudobulk predictors for predicting response to NAC alone (**D**) or in combination with ICB therapy (**E**) from Zhang et al., and for predicting T-cell clonotype expansion following treatment with NAC and ICB therapy from Bassez et al. (**F**). AUC stand for the area under the receiver operating characteristics curve. 'Random' denotes a random predictor (AUC = 0.5).

bioRxiv preprint doi: https://doi.org/10.1101/2024.06.14.598770; this version posted July 10, 2025. The copyright holder for this preprint (which was not certified by peer review) is the author/funder. This article is a US Government work. It is not subject to copyright under 17 USC 105 and is also made available for use under a CC0 license.

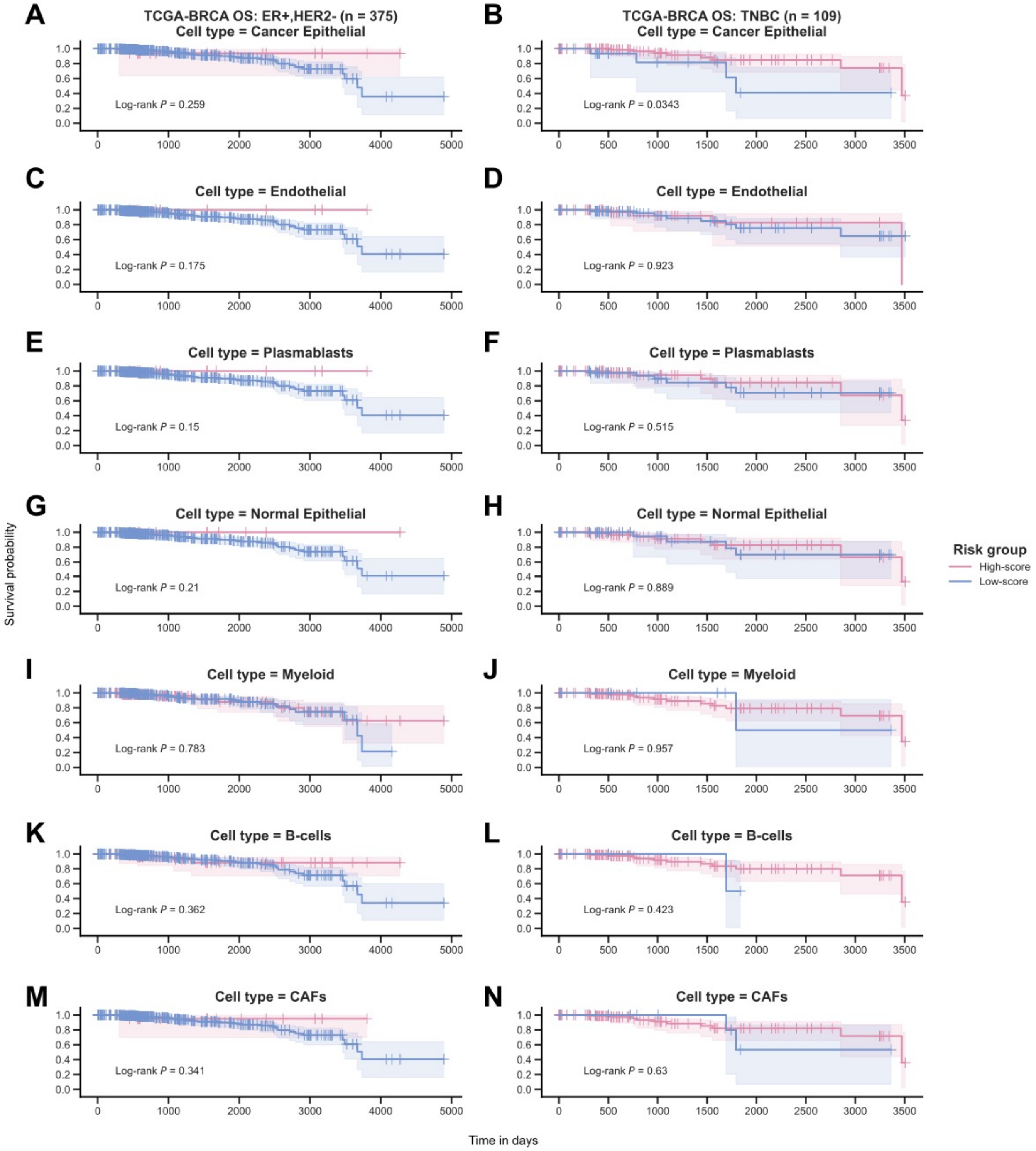

Supplementary Figure 8: The generalizability of DECODEM to the stratification of TCGA-BRCA overall survival (OS).

A-N. Kaplan-Meier curves depicting OS of early stage TCGA-BRCA patients with ER+,HER2- BC (**left**) and TNBC (**right**), stratified by DECODEM scores for seven prominent cell types. Patients were stratified into 'High-score' and 'Low-score' groups using a threshold of 0.5 on their

bioRxiv preprint doi: https://doi.org/10.1101/2024.06.14.598770; this version posted July 10, 2025. The copyright holder for this preprint (which was not certified by peer review) is the author/funder. This article is a US Government work. It is not subject to copyright under 17 USC 105 and is also made available for use under a CC0 license.

DECODEM scores for these seven cell types. The differences between the curves were computed by using a Log-rank test.

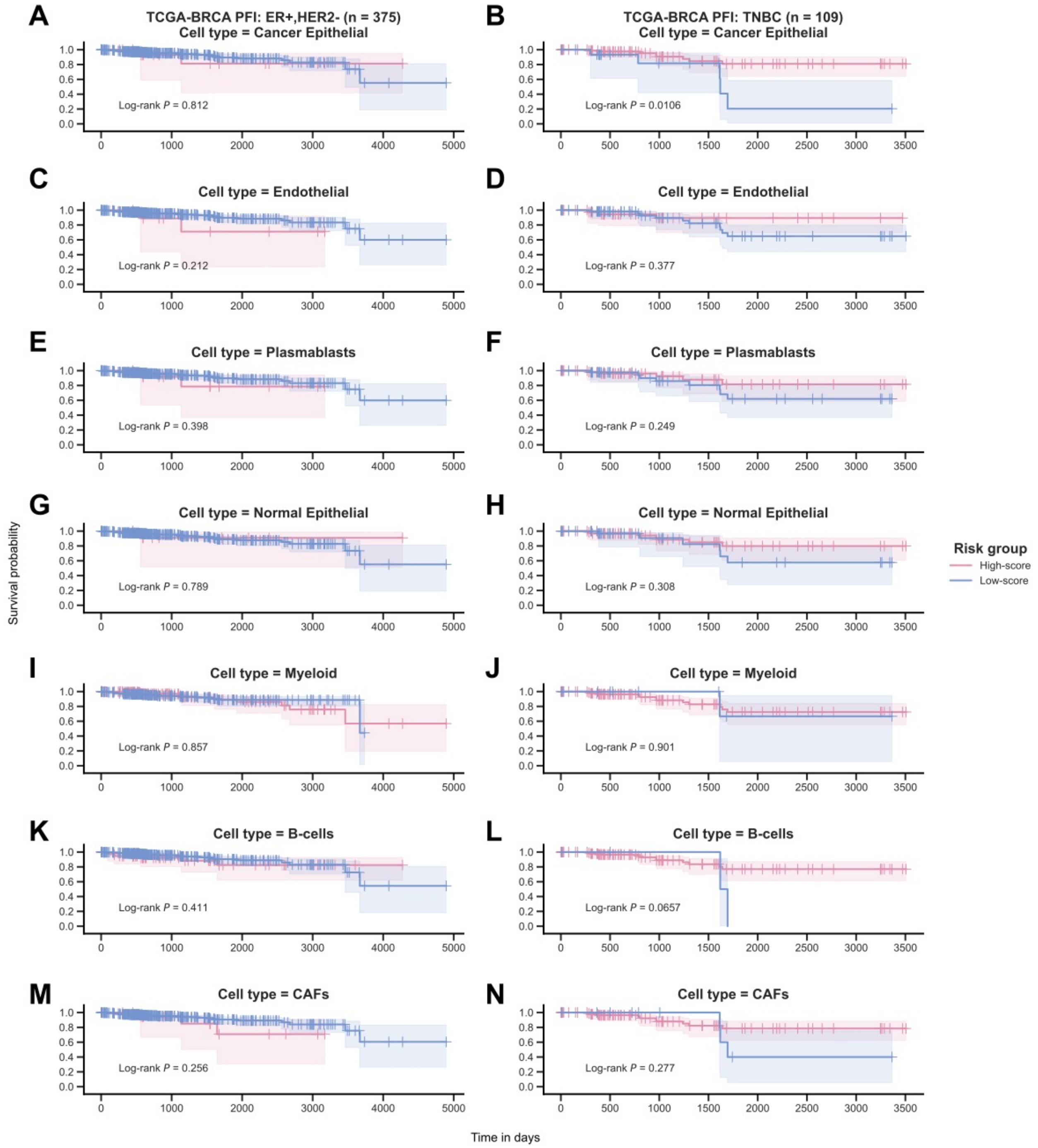

Supplementary Figure 9: The generalizability of DECODEM to the stratification TCGA-BRCA progression-free interval (PFI; synonymous to progression-free survival).

bioRxiv preprint doi: https://doi.org/10.1101/2024.06.14.598770; this version posted July 10, 2025. The copyright holder for this preprint (which was not certified by peer review) is the author/funder. This article is a US Government work. It is not subject to copyright under 17 USC 105 and is also made available for use under a CC0 license.

A-N. Kaplan-Meier curves depicting PFI of early stage TCGA-BRCA patients with ER+,HER2- BC (**left**) and TNBC (**right**), stratified by DECODEM scores for seven prominent cell types. Patients were stratified into ‘High-score’ and ‘Low-score’ groups using a threshold of 0.5 on their DECODEM scores for these seven cell types. The differences between the curves were computed by using a Log-rank test.
