# Supplementary material for: The expression patterns of different cell types and their interactions in the tumor microenvironment are predictive of breast cancer patient response to neoadjuvant chemotherapy
Source: bioRxiv. 2024 Jun 14:2024.06.14.598770. Preprint. [Version 1] doi: 10.1101/2024.06.14.598770 (PMC11451622; doi:10.1101/2024.06.14.598770)
Supplement: Supplementary file 1 [file NIHPP2024.06.14.598770v1-supplement-1.pdf]

bioRxiv preprint doi: https://doi.org/10.1101/2024.06.14.598770; this version posted June 14, 2024. The copyright holder for this preprint (which was not certified by peer review) is the author/funder. This article is a US Government work. It is not subject to copyright under 17 USC 105 and is also made available for use under a CC0 license.

SUPPLEMENTARY FIGURES

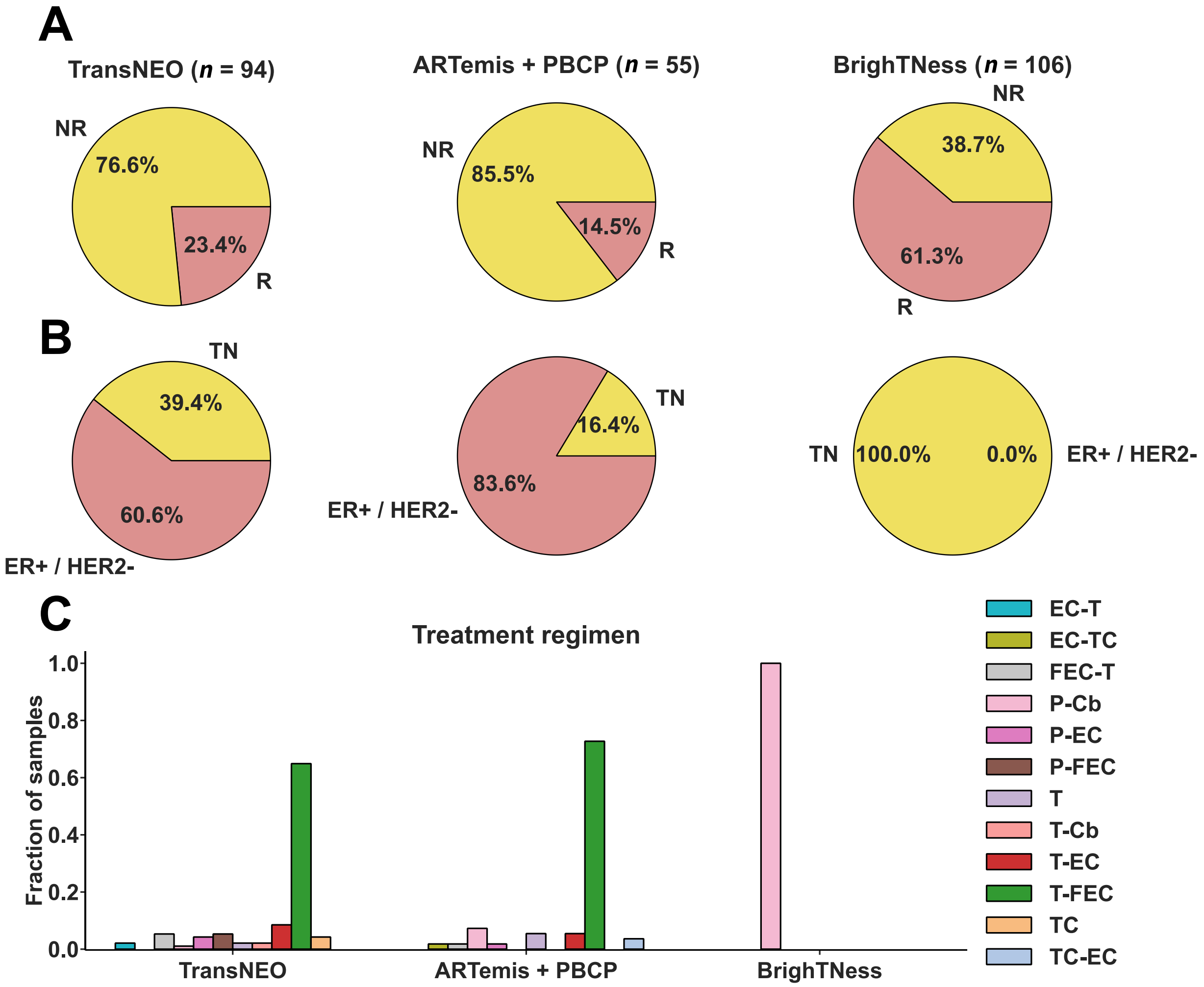

Supplementary Figure 1: Summary of the three bulk transcriptomics cohorts used.

A. Distribution of responders (R) vs. non-responders (NR) across the three cohorts. R and NR indicate the patients who achieved pCR following neoadjuvant treatment and those who did not, respectively.

B. Distribution of breast cancer subtypes across the three cohorts. ER+ / HER2- and TN stand for ER-positive, HER2-negative tumor subtype and triple negative tumor subtype, respectively.

C. Fractions of chemotherapy regimens used for neoadjuvant treatment across the three cohorts. E = Epirubicin, C = Cyclophosphamide, F = 5-Flurouracil, T = Docetaxel (Taxotere), P = Paclitaxel (Taxol), Cb = Carboplatin.

bioRxiv preprint doi: https://doi.org/10.1101/2024.06.14.598770; this version posted June 14, 2024. The copyright holder for this preprint (which was not certified by peer review) is the author/funder. This article is a US Government work. It is not subject to copyright under 17 USC 105 and is also made available for use under a CC0 license.

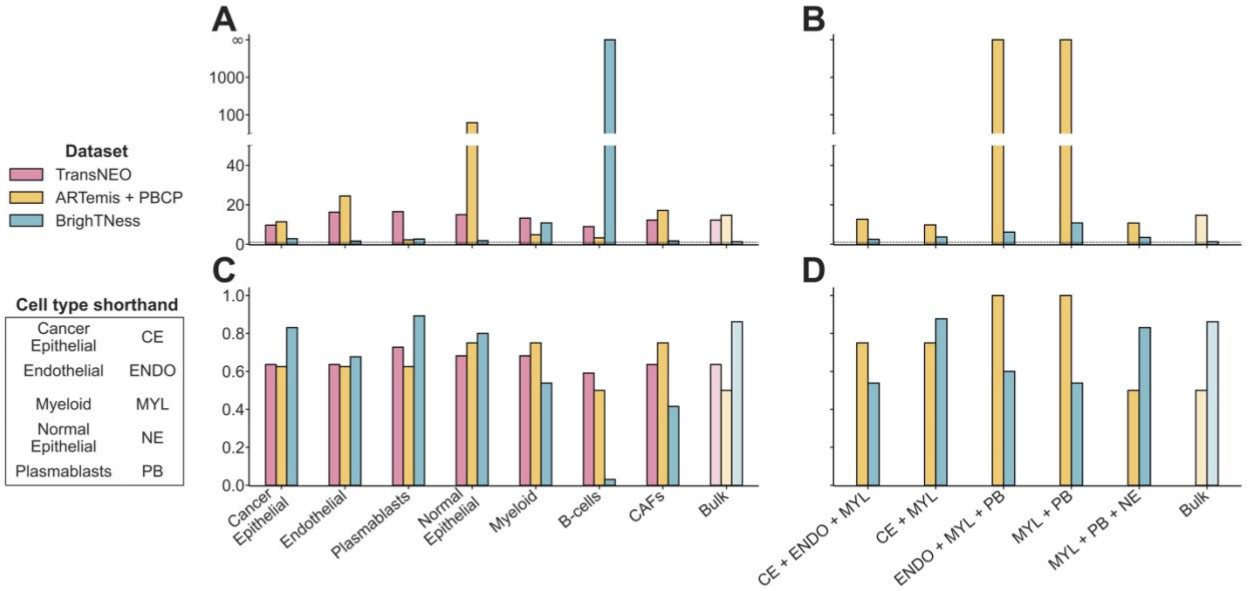

Supplementary Figure 2: The prominent cell types and ensembles mediating chemotherapy response in breast cancer tumor microenvironment.

A-B. Odds ratios for the prominent cell types (**A**) and multi-cell-type ensembles (**B**) as compared to the bulk across three cohorts. Cell types are ranked by their area under the receiver operating characteristics curve (AUC) values in cross-validation in a descending order (see **Figure 2B**). Ensembles are ranked by their AUC values in external validation in a descending order (see **Figure 3A-D**).

C-D. Sensitivities for the prominent cell types (**C**) and multi-cell-type ensembles (**D**) as compared to the bulk across the three cohorts. Cell types are ranked by their AUC values in cross-validation in a descending order (see **Figure 2B**). Cell ensembles are ranked by their AUC values in external validation in a descending order (see **Figure 3A-D**).

bioRxiv preprint doi: https://doi.org/10.1101/2024.06.14.598770; this version posted June 14, 2024. The copyright holder for this preprint (which was not certified by peer review) is the author/funder. This article is a US Government work. It is not subject to copyright under 17 USC 105 and is also made available for use under a CC0 license.

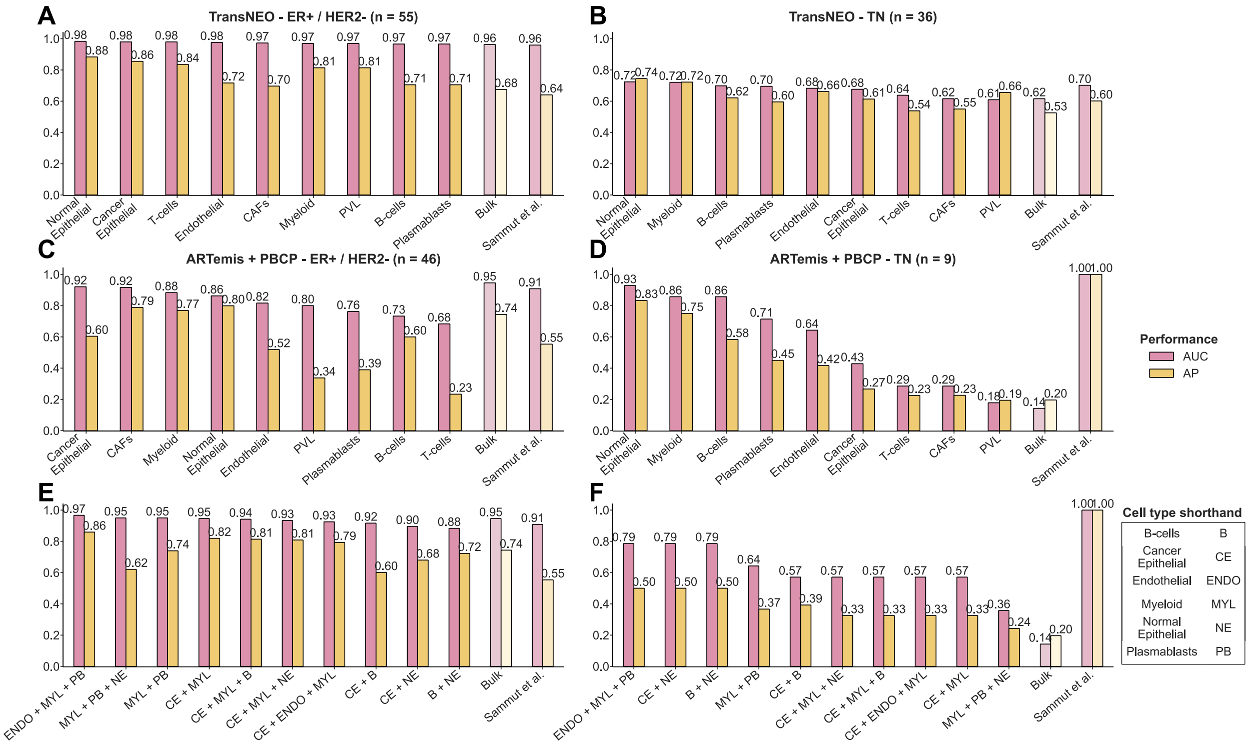

Supplementary Figure 3: The prominent cell types and ensembles mediating chemotherapy response in breast cancer tumor microenvironment in a subtype-specific manner.

A-B. Comparison of model performances in cross-validation with TransNEO for the ER-positive, HER2-negative (ER+ / HER2-; **A**) and triple negative (TN; **B**) subtypes across nine cell-type-specific, bulk, and Sammut et al. predictors. AUC and AP stand for the area under the receiver operating characteristics curve and average precision (equivalent to the area under precision-recall curve), respectively. Cell types are ranked by their AUC values in a descending order.

C-D. Comparison of model performances in external validation with ARTemis + PBCP for the ER+ / HER2- (**C**) and TN (**D**) subtypes across nine cell-type-specific, bulk, and Sammut et al. predictors. Cell types are ranked by their AUC values in a descending order.

E-F. Comparison of model performances in external validation with ARTemis + PBCP for the ER+ / HER2- (**E**) and TN (**F**) subtypes across five most prominent two-cell-ensembles, five most prominent three-cell-ensembles, bulk, and Sammut et al. predictors. Ensembles are ranked by their AUC values in a descending order.

bioRxiv preprint doi: https://doi.org/10.1101/2024.06.14.598770; this version posted June 14, 2024. The copyright holder for this preprint (which was not certified by peer review) is the author/funder. This article is a US Government work. It is not subject to copyright under 17 USC 105 and is also made available for use under a CC0 license.

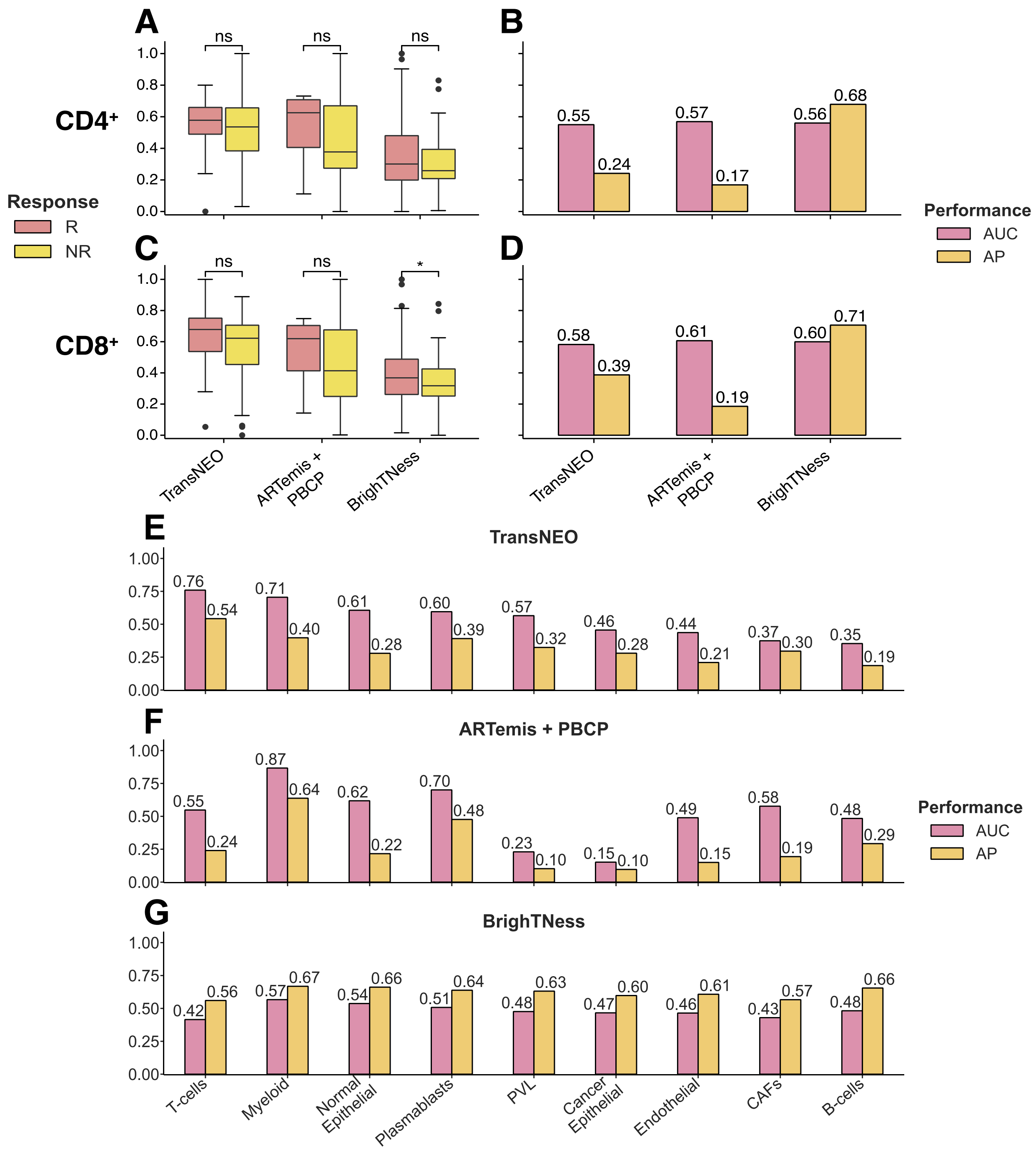

Supplementary Figure 4: The CD4⁺ / CD8⁺ T-cells enrichment and cell abundance – clinical response association analyses reaffirm the poor chemotherapy response stratification by T-cells at the current resolution.

A-B. GSVA enrichment scores (**A**) and predictive performance (**B**) of CD4⁺ T-cells for chemotherapy response stratification across TransNEO (n = 94), ARTemis + PBCP (n = 55), and BrighTNess (n =

bioRxiv preprint doi: https://doi.org/10.1101/2024.06.14.598770; this version posted June 14, 2024. The copyright holder for this preprint (which was not certified by peer review) is the author/funder. This article is a US Government work. It is not subject to copyright under 17 USC 105 and is also made available for use under a CC0 license.

106) cohorts. R and NR stand for responders and non-responders, respectively. The differences between the prediction scores were computed by using a one-tailed Wilcoxon rank-sum test. AUC and AP stand for the area under the receiver operating characteristics curve and average precision (equivalent to the area under precision-recall curve), respectively.

C-D. GSVA enrichment scores (**C**) and predictive performance (**D**) of CD8^{+} T-cells for chemotherapy response stratification across the three cohorts.

E-G. Predictive performance of abundances of the nine cell types for TransNEO (**E**), ARTemis + PBCP (**F**), and BrighTNess (**G**) cohorts. Cell types are ranked by their AUC values for TransNEO in a descending order.

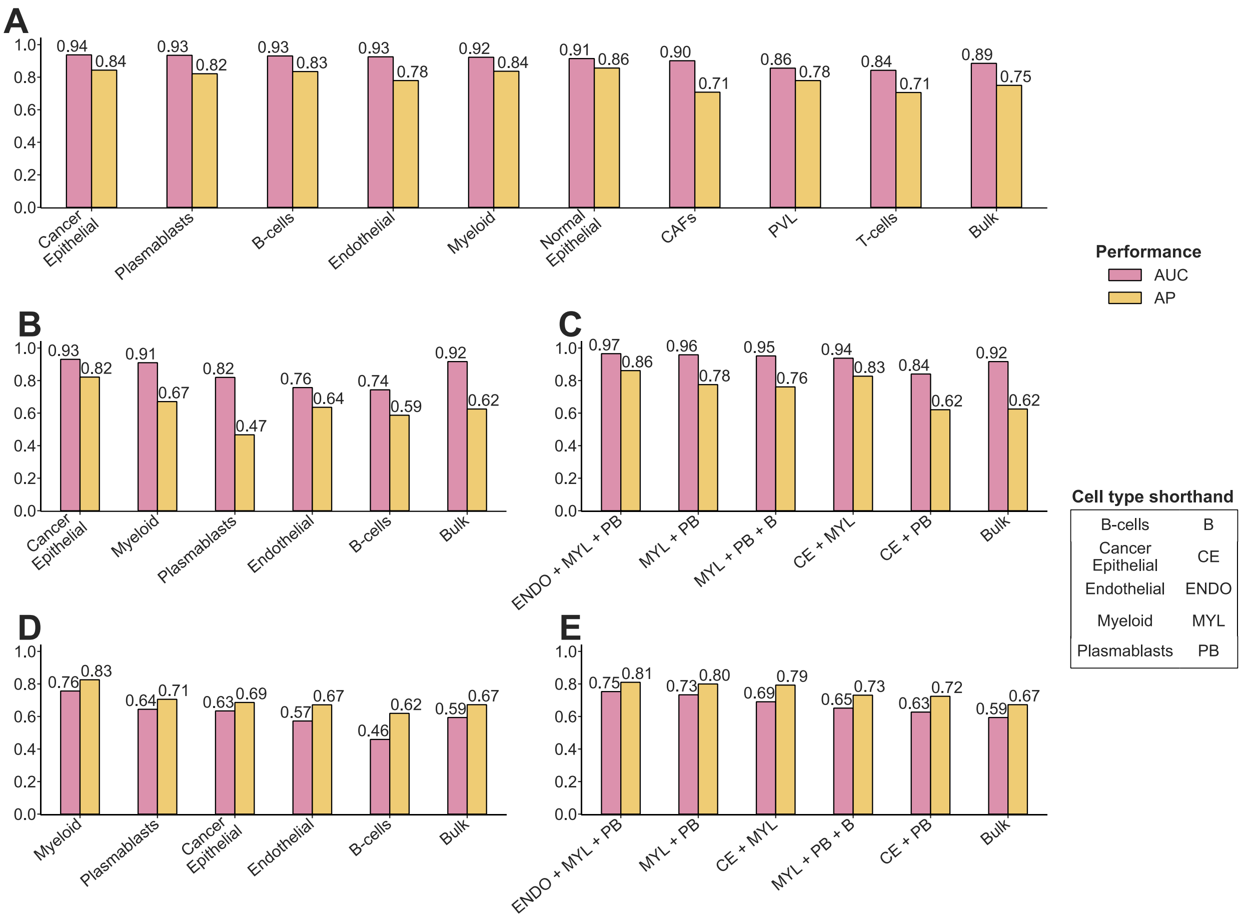

Supplementary Figure 5: The prominent immune cell types and ensembles mediating response to the most prevalent immunogenic chemotherapy regimen in each breast cancer cohort.

bioRxiv preprint doi: https://doi.org/10.1101/2024.06.14.598770; this version posted June 14, 2024. The copyright holder for this preprint (which was not certified by peer review) is the author/funder. This article is a US Government work. It is not subject to copyright under 17 USC 105 and is also made available for use under a CC0 license.

A. Comparison of model performances in cross-validation with TransNEO for the T-FEC regimen (*n* = 61) across nine cell-type-specific and bulk predictors. AUC and AP stand for the area under the receiver operating characteristics curve and average precision (equivalent to the area under precision-recall curve), respectively. Cell types are ranked by their AUC values in a descending order. Here, T = docetaxel/taxotere, F = 5-flurouracil, E = epirubicin, and C = cyclophosphamide.

B-C. Comparison of model performances in external validation with ARTemis + PBCP for the T-FEC regimen (*n* = 40) across the top cell-type-specific models (**B**) and top multi-cell-type ensembles (**C**). Cell types and ensembles are ranked by their AUC values in a descending order.

D-E. Comparison of model performances in external validation with BrighTNess for paclitaxel + carboplatin regimen (*n* = 106) across the top cell-type-specific models (**D**) and top multi-cell-type ensembles (**E**). Cell types and ensembles are ranked by their AUC values in a descending order.

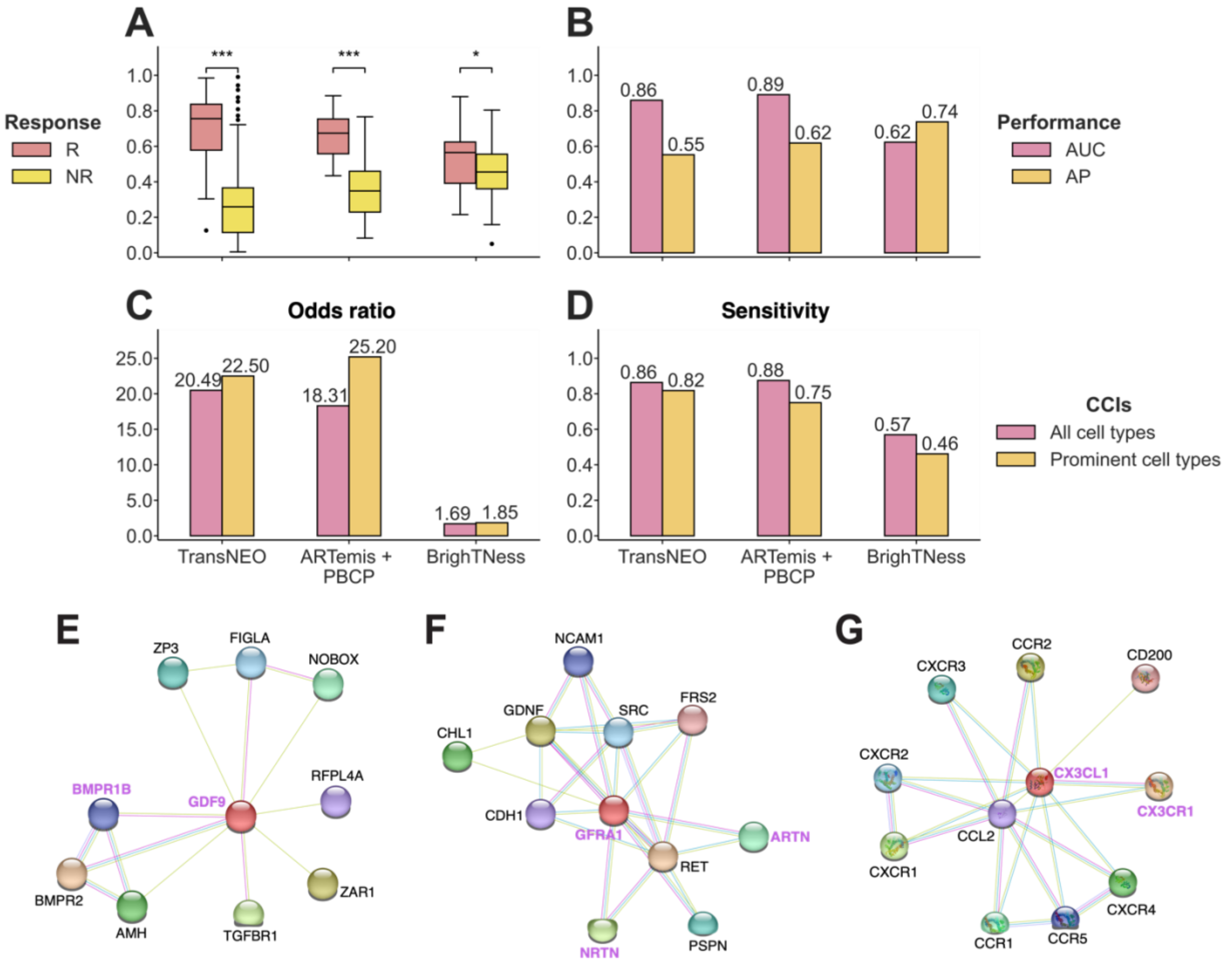

bioRxiv preprint doi: https://doi.org/10.1101/2024.06.14.598770; this version posted June 14, 2024. The copyright holder for this preprint (which was not certified by peer review) is the author/funder. This article is a US Government work. It is not subject to copyright under 17 USC 105 and is also made available for use under a CC0 license.

Supplementary Figure 6: The cell-cell interactions (CCIs) encompassing prominent cell types capture the performance of CCIs using all nine cell types in mediating chemotherapy response and are validated in single-cell (SC) breast cancer tumor microenvironment.

A-B. Prediction scores (**A**) and model performance (**B**) for DECODEMi using all available CCIs in cross-validation with TransNEO (*n* = 94) and in external validation with ARTemis + PBCP (*n* = 55) and BrighTNess (*n* = 106). R and NR stand for responders and non-responders, respectively. The differences between the prediction scores were computed by using a one-tailed Wilcoxon rank-sum test. AUC and AP stand for the area under the receiver operating characteristics curve and average precision (equivalent to the area under precision-recall curve), respectively.

C-D. Odds ratios (**C**) and sensitivities (**D**) for DECODEMi using different sets of CCIs in cross-validation with TransNEO (*n* = 94) and in external validation with ARTemis + PBCP (*n* = 55) and BrighTNess (*n* = 106).

E-G. Protein-protein interaction networks obtained from STRING [48] displaying the known interaction networks for three prominent CCIs *i.e.*, *GDF9 – BMPR1B* (**E**), *NRTN – GFRA1* and *ARTN – GFRA1* (**F**), and *CX3CL1 – CX3CR1* (**G**).

bioRxiv preprint doi: https://doi.org/10.1101/2024.06.14.598770; this version posted June 14, 2024. The copyright holder for this preprint (which was not certified by peer review) is the author/funder. This article is a US Government work. It is not subject to copyright under 17 USC 105 and is also made available for use under a CC0 license.

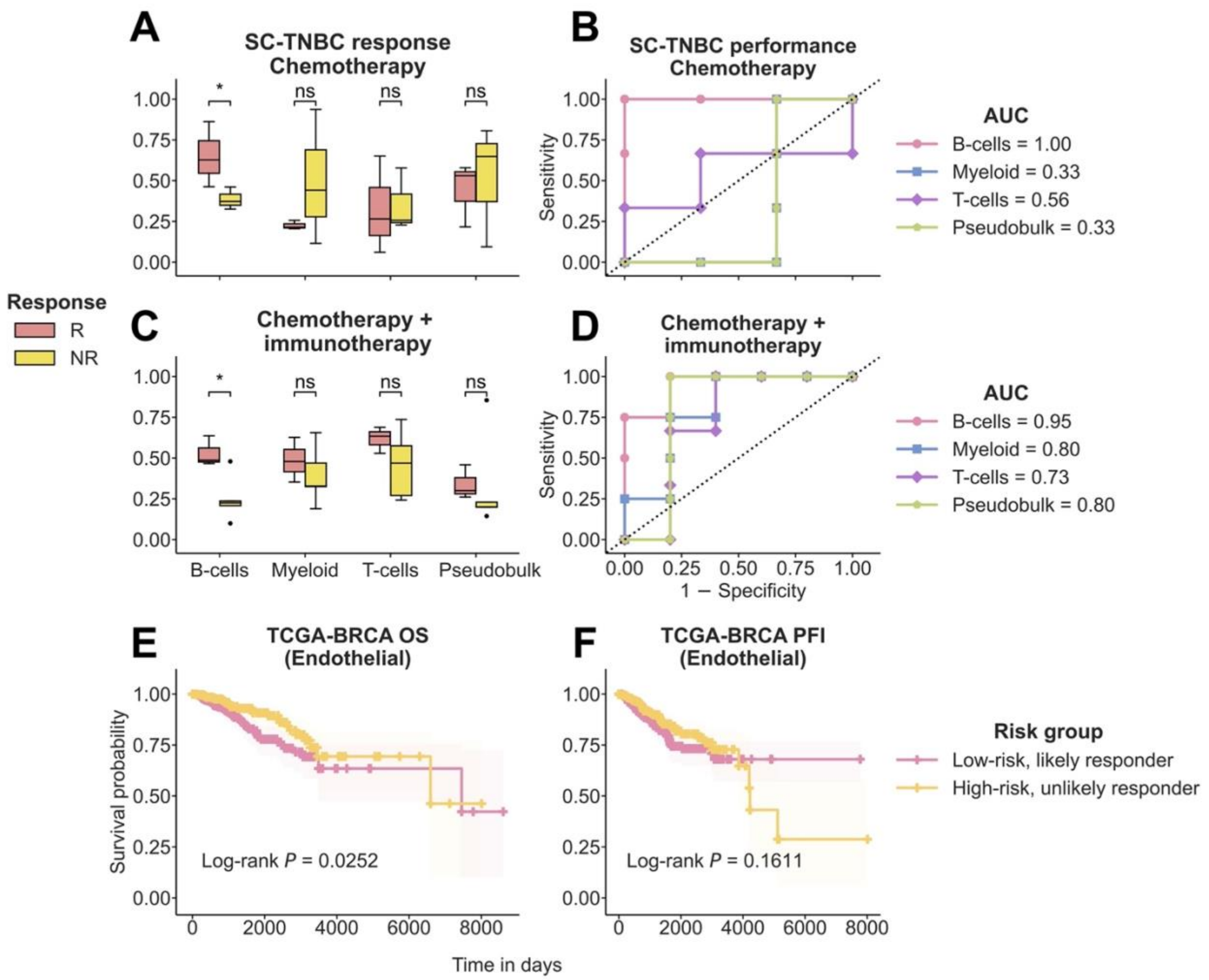

Supplementary Figure 7: The generalizability of DECODEM to single-cell (SC) transcriptomics and TCGA patient survival stratification.

A-B. Prediction scores (**A**) and the area under the receiver operating characteristics curve (AUC) values (**B**) for all available cell types in external validation with SC expression of TNBC patients (SC-TNBC) treated with chemotherapy (n = 6) from Zhang et al. [48]. Pseudobulk is shown as a baseline and computed as the mean expression across all available cells per patient after filtering for low-variance genes (threshold = 0.8). R and NR stand for responders and non-responders, respectively. The differences between the prediction scores were computed by using a one-tailed Wilcoxon rank-sum test.

C-D. Prediction scores (**C**) and the AUC values (**D**) for four cell types in external validation with SC expression of TNBC patients (SC-TNBC) treated with chemotherapy and immunotherapy (n = 9) from Zhang et al. [48].

bioRxiv preprint doi: https://doi.org/10.1101/2024.06.14.598770; this version posted June 14, 2024. The copyright holder for this preprint (which was not certified by peer review) is the author/funder. This article is a US Government work. It is not subject to copyright under 17 USC 105 and is also made available for use under a CC0 license.

E-F. Kaplan-Meier curves depicting the overall survival (OS; **E**) and progression-free interval (PFI; **F**) of the 705 BC patients from TCGA (TCGA-BRCA). Patients were stratified into low-risk, likely responder (*n* = 353) and high-risk, unlikely responder (*n* = 352) groups by their median DECODEM score computed using the expression of endothelial cells, whereby the low-risk and high-risk groups comprised individuals with scores above and below the median, respectively. The differences between the two curves were computed by using a Log-rank test.
